# The Tet-on system for controllable gene expression in the rock-inhabiting black fungus *Knufia petricola*

**DOI:** 10.1007/s00792-024-01354-2

**Published:** 2024-08-06

**Authors:** Eileen A. Erdmann, Antonia K. M. Brandhorst, Anna A. Gorbushina, Julia Schumacher

**Affiliations:** 1 Bundesanstalt für Materialforschung und -prüfung (BAM), Berlin, Germany; 2 Freie Universität Berlin, Berlin, Germany

**Keywords:** Microcolonial fungi, Inducible promoter, Bimolecular fluorescence complementation, 2A peptide, CRISPR/Cas9-mediated genome editing, Pigment synthesis

## Abstract

*Knufia petricola* is a black fungus that colonizes sun-exposed surfaces as extreme and oligotrophic environments. As ecologically important heterotrophs and biofilm-formers on human-made surfaces, black fungi form one of the most resistant groups of biodeteriorating organisms. Due to its moderate growth rate in axenic culture and available protocols for its transformation and CRISPR/Cas9-mediated genome editing, *K.* *petricola* is used for studying the morpho-physiological adaptations shared by extremophilic and extremotolerant black fungi. In this study, the bacteria-derived tetracycline (TET)-dependent promoter (Tet-on) system was implemented to enable controllable gene expression in *K. petricola*. The functionality i.e., the dose-dependent inducibility of TET-regulated constructs was investigated by using GFP fluorescence, pigment synthesis (melanin and carotenoids) and restored uracil prototrophy as reporters. The newly generated cloning vectors containing the Tet-on construct, and the validated sites in the *K.* *petricola* genome for color-selectable or neutral insertion of expression constructs complete the reverse genetics toolbox. One or multiple genes can be expressed on demand from different genomic loci or from a single construct by using 2A self-cleaving peptides, e.g., for localizing proteins and protein complexes in the *K.* *petricola* cell or for using *K.* *petricola* as host for the expression of heterologous genes.

**Supplementary Information:**

The online version contains supplementary material available at 10.1007/s00792-024-01354-2.

## Introduction

Black fungi represent a polyphyletic group of melanized Ascomycetes with members in the Arthoniomycetes, Dothideomycetes and Eurotiomycetes (Selbmann et al. 2020). They share distinct morpho-physiological adaptations such as slow growth, simple life cycles and the synthesis of protective metabolites, to resist or even propagate in natural and human-made extreme environments with regard to temperature, salinity, pH, radiation, water and nutrient availability (Prenafeta-Boldú et al. 2022; Tesei 2022; Gostincar et al. 2023). Black yeast-like fungi, often found in hypersaline and glacial habitats as well as in dishwashers, include pathogenic and opportunistic species (Gümral et al. 2015; Gostincar and Gunde-Cimerman 2023). Microcolonial growth is typical for rock- or material-inhabiting species. The thick-walled heavily melanized cells reproduce by yeast-like budding or meristematic growth and form compact black colonies on desert rocks, monuments, roofs, and photovoltaic panels (Staley et al. 1982; Wollenzien 1995; Selbmann et al. 2015; Knabe and Gorbushina 2018; Martin-Sanchez et al. 2018; Ruibal et al. 2018; Liu et al. 2022).

The rock inhabitant *Knufia petricola* (syn. *Sarcinomyces petricola*; Eurotiomycetes, Chaetothyriales) contributes to the color change and biodeterioration of antique marble in the Mediterranean (Gorbushina et al. 1993; Wollenzien et al. 1997; Gorbushina and Broughton 2009). Besides the black DHN (1,8-dihydroxynapththalene) melanin, the fungus produces carotenoids, mycosporines and extracellular polymeric substances (EPS) (Volkmann et al. 2003; Breitenbach et al. 2018; Flieger et al. 2018). The genes encoding the key enzymes for DHN melanogenesis and carotenogenesis were targeted to implement and optimize CRISPR/Cas9-mediated genome editing techniques, as their mutation results in screenable phenotypes. Thus, the mutation of *pks1* abolishes melanization making the pinkish carotenoids visible, while the simultaneous mutation of *pks1* and *phs1* or *phd1* results in albino colonies (Voigt et al. 2020). These mutants were studied regarding the dissolution of olivine, the penetration of carbonate substrates and the composition of the EPS (Gerrits et al. 2020; Tonon et al. 2021; Breitenbach et al. 2022). Further, *pks1* alone or together with *phs1* is used for the targeted integration of expression constructs into the *K.* *petricola* genome (black-pink or black-white transformant screening). For the neutral integration of expression constructs, two intergenic regions (*igr1*/*igr2*) were validated. The color-selectable integration is favorable for localization and protein–protein interactions studies using fluorescent proteins, while the intergenic regions are to be used in particular for the genetic complementation of deletion mutants (Erdmann et al. 2022). Taken together, tools for the targeted integration of several expression constructs, including five different resistance cassettes, well-working constitutive promoters and reporter genes combined in highly convenient cloning vectors, are available for *K.* *petricola*. However, a promoter for regulable gene expression is missing. Neither the promoter of the nitrate reductase-encoding gene nor the promoter of the galactokinase-encoding gene mediated gene expression in response to nitrate or galactose (Erdmann et al. 2022). This observation may point to a differently regulated primary metabolism in *K.* *petricola* as adaptation to the oligotrophic environment. Therefore, a synthetic system allowing for controllable gene expression independent of endogenous regulatory networks was envisaged.

The regulatory components from the bacterial transposon Tn10 mediating the resistance towards tetracycline (TET) were used to build an inducible promoter system for eukaryotic cells as reviewed by Berens and Hillen (2003) and Kluge et al. (2018). In brief, it is based on the tetracycline repressor protein (tetR) and the corresponding operator sequence (*tetO*) of *Escherichia* *coli*. TetR represses transcription by binding to *tetO* in the absence of TET and dissociate from *tetO* if TET is present. For using this negatively regulated circuit, the prokaryotic tetR was fused to the transcriptional activation domain from herpes simplex virus protein 16 (VP16) yielding the TET-regulated transcriptional activator (tTA). A minimal TATA-box-containing promoter was combined with one to seven copies of *tetO* to generate tTA-responsive promoters (P*tet*). Because TET prevents binding of tTA to P*tet* and therefore hinders gene expression, the system is called Tet-off. For extending the applicability of the regulatory system, binding properties of tTA were reversed (rtTA) to enable the binding to *tetO* in the presence of TET. Different rtTA variants were generated to optimize the expression in eukaryotic cells and increase the sensitivity to the TET derivate doxycycline (DOX). The resulting Tet-on system allows for the inducible gene expression by addition of TET or DOX. Taken together, the choice of (r)tTA determines if the system is positively or negatively regulated. Constructs used in filamentous Ascomycetes including *Aspergillus* species (Vogt et al. 2005; Meyer et al. 2011; Helmschrott et al. 2013; Dümig and Krappmann 2015; Sasse et al. 2016; Wanka et al. 2016; Geib and Brock 2017; Grau et al. 2018; Peng et al. 2018; Zheng et al. 2022), *Penicillium oxalicum* (Jiang et al. 2016), and *Fusarium fujikuroi* (Janevska et al. 2017; Marente et al. 2020) differ with regard to the (r)tTA expression cassette (different constitutive promoters and/or coding sequences) and P*tet* (*tetO* copy numbers and/or minimal promoters).

This study reports on the implementation of the Tet-on system for regulable gene expression in the oligotrophic fungus *K.* *petricola*. The inducer doxycycline (DOX) did not significantly impair growth and mediated the expression of genes from a synthetic P*tet* in a dose-dependent manner. This was demonstrated for *gfp*, two fusion genes from different genomic loci for localization of a protein complex, and endogenous biosynthetic genes that were fused by a viral 2A motif.

## Materials and methods

### Fungal strains and cultivation conditions

*Knufia petricola* strain A95 (CBS 123872), isolated in Athens, Greece (Gorbushina et al. 2008; Nai et al. 2013), was used as wild type (WT) (Table S1). Relevant genes and genomic regions are listed in Table S2. WT:A95 and its derivatives were cultivated on solidified or in liquid media at 25 °C (and 100 rpm) in darkness. Media contained 2% kobe agar (AppliChem) for solidification. Malt extract broth/agar [MEB/MEA: 2.0% glucose, 0.1% casein peptone (Carl Roth), 2.0% malt extract (Carl Roth)] was used as complex medium for strain maintenance. Synthetic-defined nitrate glucose broth/agar [SDNG: 0.17% BD Difco Yeast Nitrogen Base without Amino acids and Ammonium Sulfate (Thermo Fisher Scientific), 0.3% NaNO_3_, 2.0% glucose] was used as basal synthetic medium. For cultivation of auxotrophic strains 50 mg/l adenine hemisulfate (ADE, Sigma-Aldrich) or 50 mg/l uracil (URA, Sigma-Aldrich) were added. For inoculation of liquid cultures and growth assays, cells were taken from surface-grown colonies, resuspended in 1 × phosphate-buffered saline [PBS: 137 mM NaCl, 2.7 mM KCl, 10 mM Na_2_HPO_4_, 1.8 mM KH_2_PO_4_, pH 7.4], and dispersed using glass beads (3 to 5 mm) and a mixer mill (Retsch) for 5 min at 30 Hz. Cell titers were determined using a Thoma cell counting chamber. For comparative growth assays, cell titers were adjusted with PBS to 1 × 10^6^ cells/ml and dilution series down to 1 × 10^3^ cells/ml were prepared. 10-µl were dropped onto solidified media. Liquid cultures were inoculated with 1 × 10^7^ cells and incubated at 25 °C and 100 rpm. Media were supplemented with doxycycline hyclate (DOX) (Merck) from stock solutions of 25 or 50 mg/ml H_2_O. *Saccharomyces cerevisiae* FY834 (Winston et al. 1995) and the generated derivates for episomal gene expression (Table S3) were cultivated in YPD (yeast peptone dextrose; Formedium) or synthetic defined medium without uracil [SD-URA: 0.17% BD Difco Yeast Nitrogen Base without Amino acids and Ammonium Sulfate (Thermo Fisher Scientific), 0.5% (NH₄)₂SO₄, 0.077% –Ura Dropout Supplement (Takara Bio)] with 2% glucose (SD/GLU) or 4% galactose (SD/GAL) at 30 °C (and 200 rpm) in darkness.

### Standard molecular methods

Genomic DNA from *K.* *petricola* was prepared according to Voigt et al. (2020). DNA was mixed with Midori Green Direct (Biozym Scientific) and separated in 1–2% agarose gels using the 1 kb Plus DNA Ladder (New England Biolabs, NEB) as size standard. Gel electrophoresis was carried out in Mupid exU chambers and 0.5% tris–acetate-EDTA (TAE) buffer. Nucleic acids were visualized with the ChemiDoc XRS + Imager equipped with Image Lab 6.0.1 (Bio-Rad Laboratories). Total RNA from *K.* *petricola* was extracted using the TRI Reagent RNA Isolation Reagent (Sigma-Aldrich) and purified using the Monarch RNA Cleanup Kit (NEB). 1 μg of total RNA was submitted to reverse-transcription (RT) using the iScript gDNA Clear cDNA Synthesis Kit (Bio-Rad Laboratories). Standard PCR reactions were performed using desalted primers from Eurofins Genomics listed in Table S4 and Table S5, the Q5 High-Fidelity DNA Polymerase (NEB) for cloning and sequencing purposes and the *Taq* DNA Polymerase (NEB) for diagnostic applications. Plasmids listed in Table S6 were assembled *in-vivo* by homologous recombination in *S.* *cerevisiae* FY843 (Oldenburg et al. 1997) or *in-vitro* using the NEBuilder HiFi DNA Assembly Master Mix (NEB). Entry plasmids for cloning expression constructs were pRS426-derived shuttle vectors containing *E. coli bla* and *S. cerevisiae URA3* for selection (Mumberg et al. 1994; Schumacher 2012; Erdmann et al. 2022). *S. cerevisiae* was transformed with the LiAc/carrier DNA/PEG method from Gietz and Schiestl (2007). Plasmid DNA from *E.* *coli* and *S. cerevisiae* was extracted with the Monarch Plasmid Miniprep Kit (NEB). Sanger sequencing was accomplished with the Mix2Seq Kit at Eurofins Genomics. Cloning procedures were supported by using SnapGene 4.0.8 (GSL Biotech).

### Targeted editing of the *K. petricola* genome

The integration of DNA sequences, e.g., into intergenic regions (*igr*) (Fig. S1), was accomplished by introducing double strand breaks (DSB) in the desired genomic regions by plasmid-based expression of Cas9 and target-specific sgRNA and providing donor DNA – expression constructs flanked by sequences homologous to the integration site – for repair of the DSB by homologous recombination (HR). Plasmids containing one to six sgRNAs were generated by using pFC902 (Nødvig et al. 2018) as template for the amplification of tRNA-sgRNA fragments and pFC332 (Nødvig et al. 2015) as entry plasmid (Table S6). Protospacer (PS) sequences of genomic regions of interest were identified with the CRISPR site finder of Geneious Prime 2023.2.1 (Biomatters). Donor DNA from plasmid DNA was generated in three different ways: (1) by amplification using primers containing ~ 75-bp-long 5’ overhangs homologous to the specific genomic regions yielding donor DNA with short-homologous (SH) sequences (compatible with all used plasmids), (2) by digesting plasmids of the pIGRXR-XXX series with a suitable restriction enzyme, e.g., *Swa*I, cutting up- and downstream of the long-homologous (LH) sequences, or (3) by amplification from pIGRXR-XXX with the primer pair *igrX*-RF-F1/*igrX*-RF-R1 binding in the respective LH sequences for generation of donor DNA with homologous sequences of medium lengths (Table S7, Fig. S2). The generation and PEG-mediated transformation of *K.* *petricola* protoplasts were performed as described previously (Noack-Schönmann et al. 2014; Erdmann et al. 2022). The cell wall lysis buffer contained 40 mg/ml VinoTaste Pro (Novozymes) and 1 mg/ml Yatalase (Takara Bio). 2 µg of circular  Cas9- and sgRNA-delivering plasmid DNA and 10 µl of each linear donor DNA were mixed with 1 × 10^6^ protoplasts. Top agar contained 50 µg/ml hygromycin B [HYG] (AppliChem), 5 µg/ml nourseothricin [NTC] (Jena Bioscience), 100 µg/ml geneticin [G418] (Sigma-Aldrich), and/or 40 µg/ml glufosinate ammonium [GFS] (ChemPUR) for selection and 50 µg/ml ADE or 50 µg/ml URA for supplementation. Biomass of growing colonies were transferred to selective media, i.e., MEA plus 25 µg/ml HYG, 5 µg/ml NTC and/or 100 µg/ml G418, or SDNG plus 40 µg/ml GFS. The targeted integration of the expression constructs in resistant transformants was detected by diagnostic PCR combining primers binding in the expression constructs with those binding up- and downstream of the homologous sequences in the *K.* *petricola* genome (Fig. S1, Fig. S3, Fig. S4, Fig. S5).

### Fluorescence microscopy

Surface-grown cells were resuspended in 200 µl of PBS by pipetting. 10 µl of these cell suspensions or liquid cultures were spotted onto objective slides, covered with cover slips, and analyzed with a Zeiss AxioImager M2m microscope. The filter set 38 HE [excitation BP 470/40, beam splitter FT 495, emission BP 525/50] and 45 TR [excitation BP 560/40, beam splitter FT 585, emission BP 630/75] were used for examination of GFP and mCherry fluorescence, respectively. Images were captured with a Zeiss AxioCam 503 mono camera. For determining GFP fluorescence intensities, samples from liquid cultures were prepared and eight to 25 images of each replicate were acquired on the same day (new samples were prepared after ten recordings the latest). The intensity mean value (average brightness of the pixels in the object) of the GFP channel was determined using the image analysis with an automated object detection of the ZEN 3.2 (blue edition) v3.2.0.0000 software (Zeiss). The output was checked manually, only data from single or budding cells in focus – randomly selected from all areas of the field of vision and derived from at least five different images – were exported for subsequent quantification. If GFP intensities were below the detection limit and not automatically determined, they were recorded manually as zero.

## Results and discussion

### Identification of applicable doxycycline concentrations

**Fig. 1:**
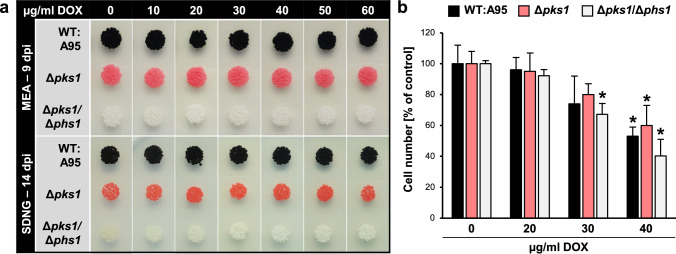
Effect of doxycycline (DOX) on growth of different *K. petricola* strains. **a** Supplementation of agar media with DOX does not affect growth. Cells of the indicated strains (10^4^, 10^3^, 10^2^, 10^1^) were dropped onto MEA or SDNG agar containing DOX in various concentrations. Colonies derived from 10^3^ cells are shown only. **b** Increasing concentrations of DOX in SDNG liquid cultures negatively affect growth. SDNG (5 ml) supplemented with DOX was inoculated with 1 × 10^7^ cells and incubated for two days at 100 rpm. Cell numbers were counted using a Thoma chamber. Mean values and standard deviations of three replicates are shown. *p ≤ 0.05 compared to untreated control (0 µg/ml DOX)

Prerequisite for using a Tet-on/-off system in *K. petricola* is that the inducer doxycycline (DOX) does not interfere with growth and pigmentation. As DOX hyclate is soluble in water, the use of a toxic solvent could be avoided. In a drop assay, the effect of varying concentrations of DOX (0 to 60 µg/ml) on the growth characteristics of the *K.* *petricola* wild type strain A95 (WT) and the non-melanized ∆*pks1* and ∆*pks1*/∆*phs1* mutants on solidified media was assessed. Malt extract medium (MEA) allows for fastest growth of *K. petricola* and is used for strain maintenance. SDNG, a synthetic medium containing nitrate and glucose as nitrogen and carbon source, respectively, as well as salts, trace elements and vitamins, is used for comparative growth assays and cultivation of strains for microscopy. Even at the highest concentration used, growth of the tested *K.* *petricola* strains on the two media was not negatively affected (Fig. 1a). In a second experiment, the effect of DOX (20, 30, 40 µg/ml) on the three strains was evaluated during cultivation in liquid SDNG medium. Cells in three cultures per strain and condition were counted after two days (Fig. 1b). The addition of 20 µg/ml DOX marginally affected cell growth. However, numbers of cells were reduced in cultures exposed to higher concentrations of DOX, which was about 20% for 30 µg/ml and 50% for 40 µg/ml. As the cell growth was equally reduced in all three strains tested, the observed effect of DOX is not related with the production of DHN melanin and/or carotenoids. Growth inhibition caused by higher concentrations of DOX was also observed in other fungi. In *A. niger* and *P. oxalicum*, reduced growth rates were reported for 125 µg/ml and 200 µg/ml DOX (Meyer et al. 2011; Jiang et al. 2016). In *F.* *fujikuroi*, the supplementation of solid medium with DOX in a concentration of 50 µg/ml DOX led to a reduction of growth by 30% (Janevska et al. 2017). In sum, DOX barely affects growth of *K.* *petricola* (at higher concentrations in liquid cultures only) allowing the use of DOX for the regulation of gene expression.

### Validation of the Tet-on system by expression of cytosolic GFP

**Fig. 2:**
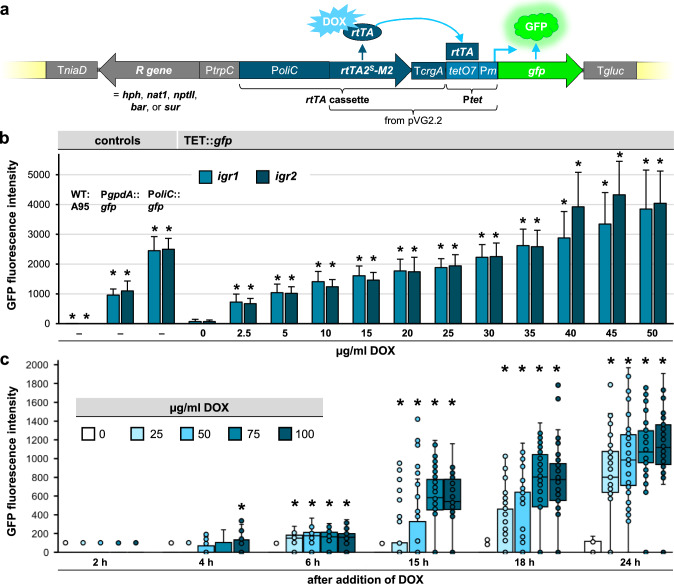
Use of GFP for validating the Tet-on system in *K. petricola*. **a** Structure of the Tet-on construct used. The core originates from pVG2.2, which was combined with P*oliC* and *gfp*::T*gluc*. The *rtTA* cassette with the *gfp* cassette was assembled with different resistance (R) cassettes and *K. petricola* sequences (*igr1*, *igr2*; yellow boxes) in pIGRXR-TGG (Fig. S2). Binding of DOX leads to a conformation change of the TET/DOX-dependent transactivator (rtTA) which induces transcription of the gene (here *gfp*) by binding to *tetO7* and activating P*min*. For validating the Tet-on system, the P*tet*::*gfp* construct was integrated in two different genomic loci (*igr1/2*) yielding strains TET::*gfp*^*igr1*^ and TET::*gfp*^*igr2*^. **b** DOX induces GFP fluorescence in *K. petricola* in a dose-dependent manner. The strains were cultivated for two days in liquid SDNG with different concentrations of DOX. GFP fluorescence intensities of TET::*gfp* cells (two transformants of TET::*gfp*^*igr1*^, n = 160; three transformants of TET::*gfp*^ *igr2*^, n = 240) and control cells (WT:A95, n = 80; P*gpdA*::*gfp*^ *igrX*^ and P*oliC*::*gfp*^*igrX*^*,* n = 320) were determined with a fixed exposure time of 40 ms. * *p* ≤ 0.001 compared to 0 µg/ml DOX. **c** GFP fluorescence becomes detectable four hours after addition of DOX. Two-day-old cells of TET::*gfp* ^*igr2*^ were exposed in one ml of liquid SDNG to five different DOX concentrations. Cells (100 µl) were submitted to fluorescence microscopy at the indicated time points. The boxplot shows the distribution of 50 data points per DOX concentration and time point (exposure time of 40 ms). * *p* ≤ 0.001 compared to values after 2 h

For implementing the Tet-on system in *K.* *petricola*, the expression construct shown in Fig. 2a was used. The core part of the construct originates from pVG2.2 (Meyer et al. 2011), a plasmid designed for DOX-regulated gene expression in *A.* *niger*. It contains the coding region of an optimized reverse TET-dependent transcriptional activator (rtTA2^S^-M2) fused to the *A. fumigatus crgA* terminator (T*crgA*) and a synthetic P*tet* (*tetO7*-P*min*). The latter comprises seven *tetO* repeats fused to the last 174 bp of the *A. nidulans gpdA* promoter (P*gpdA*). Janevska et al. (2017) constructed pNAH/N-OTGG with a P*oliC*-regulated *rtTA2*^*S*^*-M2* and P*tet*-regulated *gfp* by inserting the part from pVG2.2 between the *A. nidulans oliC* promoter (P*oliC*) and an optimized *gfp* which is fused to the *Botrytis* *cinerea gluc* terminator (T*gluc*) in pNAH/N-OGG.

The construct comprising a hygromycin resistance (hygR) cassette, and the rtTA2^S^-M2 and GFP expression cassettes was amplified with primers attaching homologous sequences to the *K.* *petricola* genome (*igr1* or *igr2*) and used together with the respective Cas9- and sgRNA-delivering plasmid for the transformation of WT:A95 protoplasts (Fig. S1). Resistant transformants were screened for the correct insertion in *igr1* or *igr2* by diagnostic PCR. Two TET::*gfp*^*igr1*^, three TET::*gfp*^*igr2*^ transformants, WT:A95 as negative control and P*gpdA*::*gfp*^*igrX*^ and P*oliC*::*gfp*^*igrX*^ strains as positive controls (constitutive promoters) were cultivated for two days in liquid SDNG containing DOX in concentrations from 0 to 50 mg/ml. Cells were resuspended and submitted to fluorescence microscopy for quantification of the GFP fluorescence intensity as indicator for gene expression (Fig. 2b). As observed before (Erdmann et al. 2022), the intensity/gene expression mediated by P*gpdA* was approx. 50% of that mediated by P*oliC*, the strongest promoter available in *K.* *petricola* so far. More importantly, GFP fluorescence was only detected in cells of the TET::*gfp*^*igrX*^ strains in the presence of DOX. The intensities increased in a concentration-dependent manner. The concentration of 5 µg/ml DOX resulted in almost the same intensity observed for P*gpdA*::*gfp*, which fits to the results observed in *A. niger* (Meyer et al. 2011). The observed intensity for P*oliC*::*gfp* was reached in TET::*gfp*^*igrX*^ strains with DOX concentrations of 30–35 µg/ml. Thus, TET::*gfp* in presence of 40–50 µg/ml DOX mediated higher gene expression than P*oliC* (approx. 150%). Standard deviations increased with the concentrations used, most likely as consequence of reduced growth in high concentrations of DOX. However, no morphological changes were observed (not shown). Taken together, the expression of *gfp* by P*tet* in *K.* *petricola* depends on DOX. The regulation does not depend on the genomic location as equal GFP fluorescence intensities were detected in strains containing the construct at *igr1* or *igr2*.

To identify minimal incubation periods and suitable concentrations of DOX to induce *gfp* expression from P*tet*, cells of one TET::*gfp*^*igr2*^ transformant were exposed for different periods to 0, 25, 50, 75 and 100 µg/ml DOX (Fig. 2c). No GFP fluorescence was detectable after two hours of incubation. The first fluorescent cells were observed after four to six hours. From fifteen hours on, very heterogenic fluorescence patterns were observed. Older and younger cells exhibited high and lower intensities, respectively (not shown). Overall, the GFP fluorescence intensities increased with the length of incubation and the concentration of DOX. These results confirm the dose-dependent expression of the reporter gene in *K.* *petricola* and provide options to avoid the long-term exposure of cells to high concentrations of DOX and to fine-tune the DOX-regulated expression of genes of interest. For instance, the incubation for 24 h in presence of 25 µg/ml may allow for significant and homogenous expression without impairing growth.

In conclusion, the used Tet-on construct is suitable for controlling the expression of *gfp* in *K.* *petricola*. Thus, pIGRXR-TGG cloning vectors containing the used Tet-on construct and different resistance cassettes and sequences for site-specific integration into the *K. petricola* genome were generated. The vectors are compatible with those of the pNXR-XXX series (Schumacher 2012) allowing the reuse of primers and the easy integration of genes of interest by in vivo recombination or in vitro DNA assembly as specified in Fig. S2. Donor DNA with long homologous sequences for insertion of expression constructs into *K.* *petricola igr1* or *igr2* can be isolated by digestion with rare-cutting restriction enzymes. Donor DNA for the insertion in any genomic locus can be generated by PCR using primers binding to the terminators of the resistance and expression cassettes and containing homologous sequences of 60 to 75 bp as 5’ overhangs.

### Additional loci for targeted integration of expression constructs

**Fig. 3:**
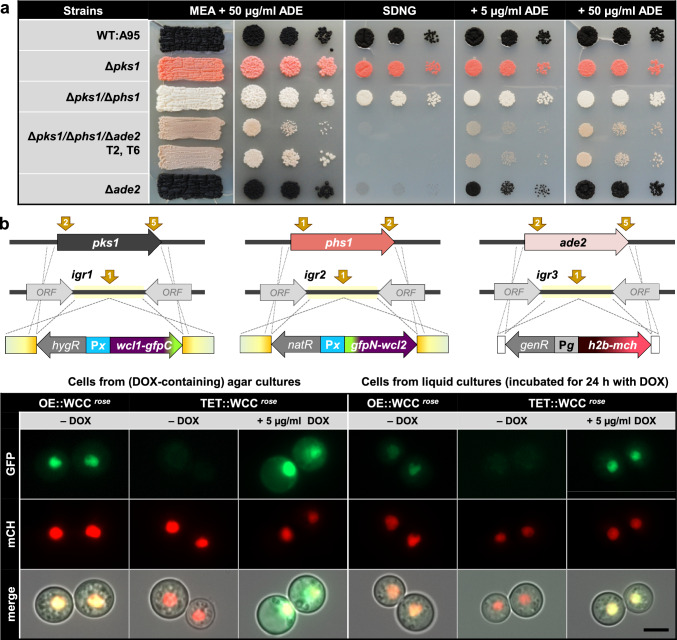
Inducible bimolecular fluorescence complementation studies in *K.* *petricola*. **a** Black-rose screening allows for the phenotypical identification of three simultaneous integration events. Cell suspensions of the indicated strains were streaked or dropped (10^4^, 10^3^, 10^2^) onto solidified media. Pictures were taken at 10 dpi (MEA) or 14 dpi (SDNG). **b** TET-regulated expression of the White collar-like transcription factors which interact in the nuclei. *K.* *petricola wcl1* and *wcl2* were fused to *gfp* fragments. P*x* was P*gpdA* for constitutive expression (OE::WCC) and P*tet* for DOX-inducible expression (TET::WCC). The two BiFC constructs and the P*gpdA* (P*g*)-controlled histone 2B-mcherry fusion construct were integrated in one step into the *K.* *petricola* genome at *igr1*, *igr2*, and *igr3* (OE::WCC ^*black*^, TET::WCC ^*black*^) or by replacing *pks1, phs1,* and *ade2* (OE::WCC ^*rose*^, TET::WCC ^*rose*^). Brown arrows indicate the Cas9 cutting sites (protospacers). Cells of strains containing the three constructs, as detected by diagnostic PCR or rose pigmentation plus resistance, were grown for three days on cellophane-covered MEA or in liquid SDNG with or without addition of DOX. Images were taken with exposure times of 100 ms for GFP and mCH. Scale bar – 5 µm

The possibility to express multiple sgRNAs along with Cas9 from a single plasmid allows for multiplexing i.e., for simultaneous genome editing events, disclosing the necessity for more insertion sites. Targeting *pks1* and *phs1* in the *K.* *petricola* genome enables the fast identification of transformants with one (black to pink) or two integrated expression constructs (black to white) in the scope of promoter or protein localization studies. In addition, microscopy of non-melanized strains is more convenient as cells must not be mechanically separated (Erdmann et al. 2022). Nonetheless, a third color-selectable insertion site was aspired to facilitate co-localization studies. *Ade2* encoding the phosphoribosylaminoimidazole carboxylase required for adenine (ADE) synthesis was considered as a candidate, as its mutation in *S. cerevisiae* results in red pigmented colonies due to the accumulation of the pathway intermediate phosphoribosylamino-imidazole (Ugolini and Bruschi 1996). The deletion of *ade2* in *K.* *petricola* resulted in ADE-auxotrophic mutants with a wild-type-like (black) pigmentation (Voigt et al. 2020). Considering, that an altered pigmentation due to Δ*ade2* may become visible when the other pigments are absent, *ade2*, *pks1* and *phs1* were simultaneously replaced by resistance cassettes (baR, natR, hygR) in the wild type background. Several whitish colonies but also few black and pink colonies appeared on the transformation plates. Light pigmented colonies were transferred to MEA supplemented with ADE and the selective agents, where few of them stayed white while most of them developed a reddish beige (rose) pigmentation. Replacement of all three genes by the respective resistance cassette were detected in chosen rose-colored transformants (not shown). For direct comparison of the growth and pigmentation characteristics of the obtained triple mutant with those of the available mutants (∆*pks1*, ∆*pks1*/∆*phs1*, ∆*ade2*) and the wild type, cells were streaked with an inoculation loop (for mimicking the step of transformant isolation) on ADE-supplemented MEA and cell suspensions were dropped onto solidified media with and without ADE (Fig. 3a). As expected, the triple mutant as the ∆*ade2* mutant failed to grow on medium lacking ADE (SDNG). On media supplemented with ADE, the triple mutants grew and exhibited a visibly altered pigmentation compared to the white ∆*pks1*/∆*phs1* and pink ∆*pks1* mutants. Therefore, the *ade2* locus is suitable – in parallel to targeting *pks1* and *phs1* – for the insertion of a third expression construct (black-rose screening). However, the ∆*pks1*/∆*phs1*/∆*ade2* mutant exhibited slightly reduced growth even on SDNG supplemented with 50 µg/ml ADE and showed an altered colony morphology in comparison to the single mutants. As this growth retardation occurred only in drop inoculation experiments, it was assumed that the ∆*pks1*/∆*phs1*/∆*ade2* cells are more sensitive to the mechanical separation step (and other stresses) rather than that their growth rate is affected.

Additional sites for the neutral insertion of expression constructs into the *K.* *petricola* genome were identified and validated as done before for *igr1* and *igr2* (Erdmann et al. 2022). Three regions on different contigs, hereafter designated as *igr3*, *igr4*, and *igr5*, were chosen as candidates as they represent shared terminator regions of two expressed genes with lengths of least 2 kb and regular GC contents (Fig. S1). Protospacers for introducing a DSB in the middle of the sequences were identified and Cas9- and sgRNA-delivering plasmids were cloned. These were used to insert a hygR-containing *gfp* expression cassette (Fig. S1b, Fig. S3). Cells from two-day-old liquid cultures of the WT:A95 and the P*oliC*::*gfp*^*igrX*^ strains were submitted to fluorescence microscopy. Similar GFP fluorescence intensities were observed for all strains containing P*oliC*::*gfp* in the five different genomic locations, while no fluorescence was detectable in wild type cells (negative control). For assessing the growth characteristics, cell suspensions of WT:A95 and the P*oliC*::*gfp*^*igrX*^ strains were spotted onto SDNG-based media. All strains showed equally reduced growth upon exposure to UV-B and on media with pH 3 or pH 8, and those supplemented with 1 M NaCl and 1 mM H_2_O_2_ for inducing osmotic and oxidative stress, respectively. The results indicate that the insertion of sequences in these regions does not interfere with growth and that genes, here *gfp*, are actively expressed from these genomic regions. Thus, five validated sites for neutral insertions and three color-selectable insertion sites are now available in *K.* *petricola* that can be used for different applications, such as for the expression of endogenous genes, reporter and fusion genes.

### Application of the Tet-on system for the expression of fusion genes

Bimolecular fluorescence complementation (BiFC) is a powerful tool for visualizing protein–protein interactions and the subcellular localization of specific protein complexes in the native cell (Kerppola 2008). However, finding promoters for appropriate expression of the two needed constructs is difficult. Native promoters may not result in detectable fluorescence signals, while the use of constitutive promoters may result in artefacts, such as the abnormal distribution in the cell and/or false positive results. These problems can be bypassed by expressing both interaction partners from the same controllable promoter.

A first BiFC approach in *K.* *petricola* demonstrated the interaction of the White collar-like transcription factors WCL1 and WCL2 in the nuclei. The formed White collar complex (WCC) is assumed to drive transcription in response to blue light (Schumacher and Gorbushina 2020). The coding regions of the *K.* *petricola* genes were fused to non-fluorescent fragments of the *B.* *cinerea* optimized *gfp* and constitutively expressed from *A.* *nidulans* P*gpdA*. The performed negative controls confirmed the specificity of the interaction (Erdmann et al. 2022). Here, the *wcl1-gfpC* and *gfpN-wcl2* fragments were inserted downstream of P*tet* in the newly constructed pIGRX-TGG vectors yielding pIGR1H-TET::W1GC and pIGR2H-TET::GNW2. In parallel, pIGR1H-OE::W1GC and pIGR2H-OE::GNW2 were generated that contain the same fragments fused to the constitutive P*gpdA*. The *wcl1-gfpC* and *gfpN-wcl2* expression constructs together with a construct for expressing a histone 2B-mcherry fusion protein were inserted either into the three pigment loci (*pks1, phs1, ade2*) or three intergenic regions (*igr1, igr2, igr3*) as specified in Fig. 3b. This resulted in four strains with a wild-type-like (black) or a rose pigmentation expressing H2B-mCH for labelling the nuclei and the two transcription factors fused to the GFP halves from P*tet* (TET) or P*gpdA* (OE). The OE::WCC ^*rose*^ and TET::WCC ^*rose*^ strains exhibited slightly impaired growth as it was observed for the rose triple knock-out strain. Therefore, the step of mechanically separating cells and high concentrations of DOX were avoided by cultivating the strains for three days on cellophane-covered MEA (0 or 5 µg/ml DOX) or in liquid SDNG medium (addition of 5 µg/ml DOX 24 h prior to microscopy). Green fluorescence in the nuclei was observed for the control strain (OE::WCC ^*rose*^) and TET::WCC ^*rose*^ exclusively in the presence of DOX. Notably, cells from MEA showed brighter GFP fluorescence than those from liquid cultivation.

Chosen black strains containing the three constructs as detected by diagnostic PCR were used to validate and optimize the induction conditions (Fig. S4). Two independent transformants of TET::WCC ^*black*^ and OE::WCC ^*black*^ were cultivated with varying DOX concentrations (0, 25, 50, 75 and 100 µg/ml). Cells were screened by fluorescence microscopy after 6, 12 and 18 h. After six hours of induction, GFP fluorescence – indicating the formation of a WCC – was observed in about two-thirds of the TET::WCC ^*black*^ cells incubated with DOX and nearly all OE::WCC ^*black*^ cells. Nuclear GFP fluorescence with roughly the same intensity compared to that of the control strain was detected after 12 h of induction with 25 and 50 µg/ml DOX. In contrast, after 18 h GFP fluorescence intensities in the TET::WCC ^*black*^ cells were much higher than those detected in OE::WC ^*black*^ cells. To conclude, the exposure of cells for 12 h to 25 µg/ml DOX in liquid SDNG was sufficient to detect explicit GFP fluorescence in the nuclei due the reconstitution of a functional GFP.

These experiments demonstrated that the used Tet-on construct is suitable for controllable BiFC studies and that it has the capacity to mediate much higher gene expression than the applied standard promoters in dependence on the inducer DOX. The strategies for simultaneous integration of three expression constructs, with or without color transformant screening, were validated indicating further that the insertion of two Tet-on constructs though the long stretches of identical sequences is feasible. Four cloning vectors with the Tet-on construct for inserting a gene of interest upstream or downstream of *gfpN* or *gfpC* were generated for further BiFC studies in *K.* *petricola* and other fungi (Fig. S2b).

### Application of the Tet-on system for the expression of biosynthetic genes

**Fig. 4:**
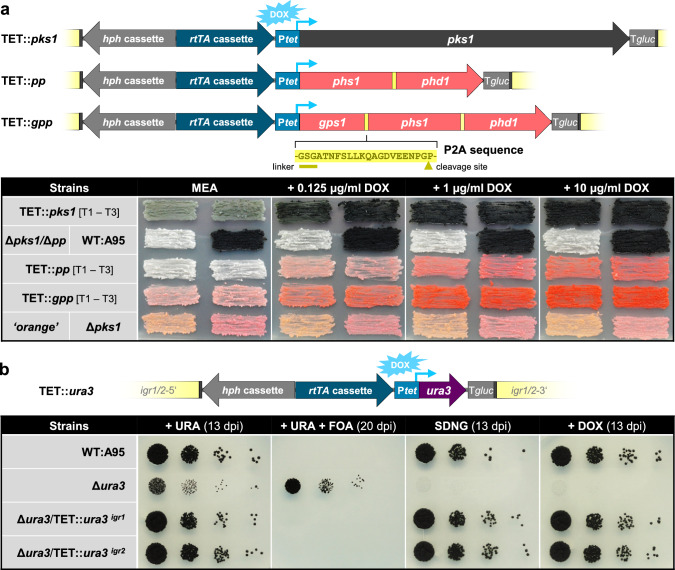
TET-regulated synthesis genes are highly inducible but show moderate basal activity in *K.* *petricola*. **a** Expression of genes for synthesis of DHN melanin and carotenoids. The viral P2A sequence was used to express multiple proteins from a polycistronic transcript. The expression constructs were inserted in *igr2* of Δ*pks1*/Δ*phs1-phd1* mutants defective in DHN melanin and carotenoid synthesis. Indicated strains were cultivated for four days on MEA supplemented with DOX as shown. PKS1 – polyketide synthase, GPS1 – geranylgeranyl pyrophosphate synthase, PHS1 – bifunctional lycopene cyclase/phytoene synthase, PHD1 – phytoene desaturase. **b** Expression of *ura3*, essential for growth on minimal medium. TET::*ura3* expression constructs were integrated at *igr1* or *igr2* in a Δ*ura3* mutant. Cells (10^4^, 10^3^, 10^2^, 10.^1^) were dropped onto SDNG, supplemented with uracil (URA, 50 µg/ml), 5-fluoroorotic acid (FOA, 1 mg/ml), or DOX (1 µg/ml)

Gene expression is an important tool, such as for studying cell biology by live cell imaging approaches and exploring biosynthetic pathways in the native and/or heterologous hosts. Usually, as done for the BiFC approach in this study, two or more genes are expressed from individual constructs in different genomic loci. However, the insertion of a viral 2A self-cleaving peptide between coding regions may enable the expression of multiple proteins from a polycistronic transcript (Kim et al. 2011; Liu et al. 2017; Schuetze and Meyer 2017). Here, the genes encoding the key enzymes for DHN melanogenesis and carotenogenesis were used as reporters to follow their expression mediated by the Tet-on system in *K.* *petricola*. Three constructs were cloned and inserted into *igr2* in the generated pigment-free recipient strain ∆*pks1*/∆*phs1-phd1* (Fig. S5): *pks1* alone (TET::*pks1*) for restoration of DHN melanogenesis, *phs1* and *phd1* linked via a P2A motif (TET::*pp*) for restoration of carotenogenesis, and *gps1*, *phs1* and *phd1* linked via P2A motifs (TET::*gpp*) for restoration and overstimulation of carotenogenesis if applicable (Fig. 4a, Fig. S6). *Phs1* and *phd1* encoding a bifunctional lycopene cyclase/phytoene synthase and a phytoene desaturase, respectively, catalyze in fungi the consecutive reactions leading to β-carotene and torulene (Avalos et al. 2017; Sandmann 2022). *Gps1* encoding a geranylgeranyl pyrophosphate synthase was included as its overexpression is known to result in higher product yields by providing more precursors (Verwaal et al. 2007; Jiao et al. 2018). Correct insertion of the constructs in *igr2* of hygR transformants was detected by diagnostic PCR (not shown). The generated strains were cultivated together with the wild type and different pigment mutants as controls on MEA without and with DOX in different concentrations (0.125, 0.25, 0.5, 1, 5, 10, 30 µg/ml). The formation of pigments by the TET strains in presence of high DOX concentrations became already visible after two to three days of incubation though not much biomass was present (not shown). After four days of incubation, all TET strains produced more pigments on MEA supplemented with DOX than without DOX. Low dosages were sufficient; the lowest concentration tested (0.125 µg/ml) resulted already in visible pigment accumulation, a more pronounced pigmentation was observed for the carotenoid gene-expressing strains on MEA supplemented with 1 µg/ml. Higher concentrations did not visibly increase pigment synthesis. The overexpression of *gps1* (native plus TET-regulated copy) caused a more reddish pigmentation, suggesting that more carotenoids were produced. The restoration of carotenoid synthesis in TET::*pp* and TET::*gpp* strains in presence of DOX indicated that the complete constructs were transcribed, the polycistronic transcripts were translated into single proteins due to functional P2A motifs causing ribosome skipping, and that the enzymes were functional despite the remaining P2A-derived residues. The Tet-on constructs allowed for expression of the carotenogenic genes in *S. cerevisiae* as well indicating at least moderate expression of rtTA mediated by *A.* *nidulans* P*oliC* (Fig. S7). Thus, the experiment demonstrated the tunable expression of the synthesis genes by DOX. However, the light gray and light pink pigmentation of TET::*pks1* and TET::*gpp* strains in absence of DOX indicated a basal activity of the Tet-on construct in *K.* *petricola* (Fig. 4a).

To take a closer look at this, a TET-regulated *ura3* was neutrally integrated in *igr1* or *igr2* in the ∆*ura3* mutant (Fig. 4b). Mutation of *ura3* encoding the orotidine 5-phosphate decarboxylase for the *de-novo* synthesis of pyrimidine ribonucleotides results in an auxotrophy for uracil (URA) and the resistance to 5-fluorootic acid (FOA) (Voigt et al. 2020). Cells of the generated ∆*ura3*/TET::*ura3*^*igrX*^ strains together with those of the wild type and the recipient strain ∆*ura3* were dropped onto SDNG (without URA) supplemented with URA, FOA or 1 µg/ml DOX to test the restoration of URA synthesis. Control strains showed the expected phenotype; wild type cells grew on all media except to SDNG with FOA whereas the ∆*ura3* mutant grew on URA- and FOA-supplemented SDNG. However, the ∆*ura3*/TET::*ura3* strains grew – without the addition of DOX – in a wild-type-like manner. Consequently, the basal activity of the Tet-on construct mediated sufficient expression of this essential nutritional gene.

Taken together, the Tet-on system together with the viral P2A motif allows for inducible (over)-expression of one or more biosynthetic genes from a single construct/genomic locus in *K.* *petricola*.

## Conclusions

As DOX is not toxic in relevant culture conditions and concentrations, it can be used to control gene expression in *K.* *petricola* by Tet-on and Tet-off systems. Here, we validated the suitability of the Tet-on construct that was developed by Meyer et al. (2011) for the application in *A.* *niger* for driving gene expression in a DOX-dependent manner in *K.* *petricola*. Two major reporter activities (GFP fluorescence and pigmentation) were studied. GFP fluorescence was detected in DOX-treated cells only. However, for the TET-regulated biosynthetic genes a basal activity of the Tet-on construct was observed that must be considered for further applications. Nevertheless, reporter genes were expressed in a dose-dependent manner (concentration and incubation time) with the possibility of higher expression levels compared to those mediated by constitutive promoters (P*oliC,* P*gpdA*) and tested endogenous promoters. The toolbox for knock-in approaches in *K.* *petricola* was extended by cloning vectors containing the Tet-on construct and different resistance cassettes, and additional integration sites in its genome. Up to three expression constructs can be inserted in the genome with color selection (*pks1*, *phs1*, *ade2*) and up to five expression constructs in intergenic regions (*igr1-5*) for leaving all genes intact. This extension allowed the targeted integration of three expression constructs at the same time for testing the simultaneous induction of two TET-regulated expression constructs for co-localizing the WCC due to a reconstituted GFP with a H2B-mcherry protein in the nuclei. By fusing two or three genes for carotenogenesis via P2A motifs, it was demonstrated that also multiple genes can be expressed in a DOX-dependent manner from a single construct/genomic locus. The simplicity to edit the genome, the possibility to express genes from synthetic clusters and convenient intrinsic characteristics render *K.* *petricola* a suitable host for the expression of heterologous genes for production of secondary metabolites or secreted enzymes. *K.* *petricola* has a very limited secondary metabolism but can bear the burden of constitutive pigment formation and protein secretion. Acetyl-CoA accumulating in white Δ*pks1*/Δ*phs1* strains can be used for other synthesis pathways and their yeast-like growth facilitates cultivation. Gene expression mediated by the Tet-on system in a metabolism/medium-independent manner further enables the design of an inducible *in-vivo* mutagenesis tool for forward genetics screens. In sum, the well-stocked toolbox for editing the genome of the black fungus *K.* *petricola* allows for elucidating the shared traits of extremophilic and extremotolerant fungi including the regulation of the primary metabolism in an oligotrophic environment, the unconventional modes of cell division as well as the genetic basis and regulation of the characteristic metabolites.

*K. petricola* serves as a model for eukaryotic heterotrophic biofilm-formers on diverse human-made surfaces – from marble monuments to renewable solar energy production facilities. As a highly adhesive and material-interacting organism (olivine dissolution, penetration of carbonate substrates and corrosive activity of its EPS was demonstrated for *K.* *petricola*), this genetically amenable black fungus offers a modern tool of highly reproducible and relevant biodeterioration research. Tools and experiences gained from *K.* *petricola* as a model system are transferrable to other challenging (black) fungi. Recently these tools have already allowed us to genetically manipulate the cryptoendolithic *Cryomyces antarcticus* (Catanzaro et al. 2024), a black fungus with extremely low growth rates and assumed as being one of the most stress-resistant eukaryotes on Earth.

## Supplementary Information

Below is the link to the electronic supplementary material.

Supplementary file1 (PDF 1848 KB)

## Data Availability

All data supporting the findings of this study are available within the paper and its Supplementary Information.

## Abbreviations

CRISPR: Clustered Regularly Interspaced Short Palindromic Repeats
Cas9: CRISPR-associated protein-9 nuclease
GFP: Green Fluorescent Protein
TET: Tetracycline
DOX: Doxycycline

## References

[CR1] Avalos J, Pardo-Medina J, Parra-Rivero O, Ruger-Herreros M, Rodriguez-Ortiz R, Hornero-Mendez D, Limon MC (2017) Carotenoid biosynthesis in Fusarium. J Fungi (Basel) 3:39. 10.3390/jof3030039 29371556 10.3390/jof3030039 PMC5715946

[CR2] Berens C, Hillen W (2003) Gene regulation by tetracyclines: constraints of resistance regulation in bacteria shape TetR for application in eukaryotes. Eur J Biochem 270:3109–3121. 10.1046/j.1432-1033.2003.03694.x 12869186 10.1046/j.1432-1033.2003.03694.x

[CR3] Breitenbach R, Silbernagl D, Toepel J, Sturm H, Broughton WJ, Sassaki GL, Gorbushina AA (2018) Corrosive extracellular polysaccharides of the rock-inhabiting model fungus *Knufia petricola*. Extremophiles 22:165–175. 10.1007/s00792-017-0984-5 29275441 10.1007/s00792-017-0984-5 PMC5847175

[CR4] Breitenbach R, Gerrits R, Dementyeva P, Knabe N, Schumacher J, Feldmann I et al (2022) The role of extracellular polymeric substances of fungal biofilms in mineral attachment and weathering. Npj Mater Degrad 6:42. 10.1038/s41529-022-00253-1

[CR5] Catanzaro I, Gerrits R, Feldmann I, Gorbushina AA, Onofri S, Schumacher J (2024) Deletion of the polyketide synthase-encoding gene pks1 prevents melanization in the extremophilic fungus Cryomyces antarcticus. IUBMB Life 2024:1–19. 10.1002/iub.2895 10.1002/iub.2895 PMC11580375 39011777

[CR6] Dümig M, Krappmann S (2015) Controlling fungal gene expression using the doxycycline-dependent Tet-ON system in Aspergillus fumigatus. In: van den Berg M, Maruthachalam K (eds) Genetic transformation systems in fungi, vol 2. Springer, Cham, pp 131–138. 10.1007/978-3-319-10503-1_10.

[CR7] Erdmann EA, Nitsche S, Gorbushina AA, Schumacher J (2022) Genetic engineering of the rock inhabitant *Knufia petricola* provides insight into the biology of extremotolerant black fungi. Front Fungal Biol 3:862429. 10.3389/ffunb.2022.862429 37746170 10.3389/ffunb.2022.862429 PMC10512386

[CR8] Flieger K, Knabe N, Toepel J (2018) Development of an improved carotenoid extraction method to characterize the carotenoid composition under oxidative stress and cold temperature in the rock inhabiting fungus Knufia petricola A95. J Fungi (basel) 4:124. 10.3390/jof4040124 30424015 10.3390/jof4040124 PMC6308947

[CR9] Geib E, Brock M (2017) ATNT: an enhanced system for expression of polycistronic secondary metabolite gene clusters in *Aspergillus niger*. Fungal Biol Biotechnol 4:13. 10.1186/s40694-017-0042-1 29270299 10.1186/s40694-017-0042-1 PMC5735947

[CR10] Gerrits R, Pokharel R, Breitenbach R, Radnik J, Feldmann I, Schuessler JA et al (2020) How the rock-inhabiting fungus *K. petricola* A95 enhances olivine dissolution through attachment. Geochim Cosmochim Acta 282:76–97. 10.1016/j.gca.2020.05.010

[CR11] Gietz RD, Schiestl RH (2007) Frozen competent yeast cells that can be transformed with high efficiency using the LiAc/SS carrier DNA/PEG method. Nat Protocols 2(1):1–4. 10.1038/nprot.2007.17 17401330 10.1038/nprot.2007.17

[CR12] Gorbushina AA, Broughton WJ (2009) Microbiology of the atmosphere-rock interface: how biological interactions and physical stresses modulate a sophisticated microbial ecosystem. Annu Rev Microbiol 63:431–450. 10.1146/annurev.micro.091208.073349 19575564 10.1146/annurev.micro.091208.073349

[CR13] Gorbushina AA, Krumbein WE, Hamman CH, Panina L, Soukharjevski S, Wollenzien U (1993) Role of black fungi in color change and biodeterioration of antique marbles. Geomicrobiol J 11:205–221. 10.1080/01490459309377952

[CR14] Gorbushina AA, Kotlova ER, Sherstneva OA (2008) Cellular responses of microcolonial rock fungi to long-term desiccation and subsequent rehydration. Stud Mycol 61:91–97. 10.3114/sim.2008.61.09 19287531 10.3114/sim.2008.61.09 PMC2610304

[CR15] Gostincar C, Gunde-Cimerman N (2023) Understanding fungi in glacial and hypersaline environments. Annu Rev Microbiol 77:89–109. 10.1146/annurev-micro-032521-020922 37001148 10.1146/annurev-micro-032521-020922

[CR16] Gostincar C, Stajich JE, Gunde-Cimerman N (2023) Extremophilic and extremotolerant fungi. Curr Biol 33:R752–R756. 10.1016/j.cub.2023.06.011 37490857 10.1016/j.cub.2023.06.011

[CR17] Grau MF, Entwistle R, Chiang YM, Ahuja M, Oakley CE, Akashi T et al (2018) Hybrid transcription factor engineering activates the silent secondary metabolite gene cluster for (+)-asperlin in *Aspergillus nidulans*. ACS Chem Biol 13:3193–3205. 10.1021/acschembio.8b00679 30339758 10.1021/acschembio.8b00679 PMC6546424

[CR18] Gümral R, Özhak-Baysan B, Tümgör A, Saraçlı MA, Yıldıran ŞT, Ilkit M et al (2015) Dishwashers provide a selective extreme environment for human-opportunistic yeast-like fungi. Fungal Divers 76:1–9. 10.1007/s13225-015-0327-8

[CR19] Helmschrott C, Sasse A, Samantaray S, Krappmann S, Wagener J (2013) Upgrading fungal gene expression on demand: improved systems for doxycycline-dependent silencing in *Aspergillus fumigatus*. Appl Environ Microbiol 79:1751–1754. 10.1128/AEM.03626-12 23275515 10.1128/AEM.03626-12 PMC3591957

[CR20] Janevska S, Arndt B, Baumann L, Apken L, Mauriz Marques L, Humpf H-U, Tudzynski B (2017) Establishment of the inducible Tet-on system for the activation of the silent trichosetin gene cluster in *Fusarium fujikuroi*. Toxins 9:126. 10.3390/toxins9040126 28379186 10.3390/toxins9040126 PMC5408200

[CR21] Jiang B, Zhang R, Feng D, Wang F, Liu K, Jiang Y et al (2016) A Tet-on and Cre-loxP based genetic engineering system for convenient recycling of selection markers in *Penicillium oxalicum*. Front Microbiol 7:485. 10.3389/fmicb.2016.00485 27148179 10.3389/fmicb.2016.00485 PMC4828452

[CR22] Jiao X, Sun W, Zhang Y, Liu X, Zhang Q, Wang Q et al (2018) Exchanging the order of carotenogenic genes linked by porcine teschovirus-1 2A peptide enable to optimize carotenoid metabolic pathway in *Saccharomyces cerevisiae*. RSC Adv 8:34967–34972. 10.1039/c8ra06510a 35547038 10.1039/c8ra06510a PMC9087642

[CR23] Kerppola TK (2008) Bimolecular fluorescence complementation (BiFC) analysis as a probe of protein interactions in living cells. Annu Rev Biophys 37:465–487. 10.1146/annurev.biophys.37.032807.125842 18573091 10.1146/annurev.biophys.37.032807.125842 PMC2829326

[CR24] Kim JH, Lee SR, Li LH, Park HJ, Park JH, Lee KY et al (2011) High cleavage efficiency of a 2A peptide derived from porcine teschovirus-1 in human cell lines, zebrafish and mice. PLoS ONE 6:e18556. 10.1371/journal.pone.0018556 21602908 10.1371/journal.pone.0018556 PMC3084703

[CR25] Kluge J, Terfehr D, Kück U (2018) Inducible promoters and functional genomic approaches for the genetic engineering of filamentous fungi. Appl Microbiol Biotechnol 102:6357–6372. 10.1007/s00253-018-9115-1 29860590 10.1007/s00253-018-9115-1 PMC6061484

[CR26] Knabe N, Gorbushina AA (2018) Territories of rock-inhabiting fungi: survival on and alteration of solid air-exposed surfaces. In: Gurtler V, Trevors JT (eds) Microbiology of atypical environments. Elsevier, pp 145–169. 10.1016/bs.mim.2018.06.001

[CR27] Liu Z, Chen O, Wall JBJ, Zheng M, Zhou Y, Wang L et al (2017) Systematic comparison of 2A peptides for cloning multi-genes in a polycistronic vector. Sci Rep 7:2193. 10.1038/s41598-017-02460-2 28526819 10.1038/s41598-017-02460-2 PMC5438344

[CR28] Liu B, Fu R, Wu B, Liu X, Xiang M (2022) Rock-inhabiting fungi: terminology, diversity, evolution and adaptation mechanisms. Mycology 13:1–31. 10.1080/21501203.2021.2002452 35186410 10.1080/21501203.2021.2002452 PMC8856086

[CR29] Marente J, Avalos J, Limon MC (2020) Controlled transcription of regulator gene carS by Tet-on or by a strong promoter confirms its role as a repressor of carotenoid biosynthesis in Fusarium fujikuroi. Microorganisms 9:71. 10.3390/microorganisms9010071 33383912 10.3390/microorganisms9010071 PMC7824685

[CR30] Martin-Sanchez PM, Gebhardt C, Toepel J, Barry J, Munzke N, Günster J, Gorbushina AA (2018) Monitoring microbial soiling in photovoltaic systems: a qPCR-based approach. Int Biodeterior Biodegrad 129:13–22. 10.1016/j.ibiod.2017.12.008

[CR31] Meyer V, Wanka F, van Gent J, Arentshorst M, van den Hondel CA, Ram AF (2011) Fungal gene expression on demand: an inducible, tunable, and metabolism-independent expression system for *Aspergillus niger*. Appl Environ Microbiol 77:2975–2983. 10.1128/AEM.02740-10 21378046 10.1128/AEM.02740-10 PMC3126388

[CR32] Mumberg D, Müller R, Funk M (1994) Regulatable promoters of *Saccharomyces cerevisiae*: comparison of transcriptional activity and their use for heterologous expression. Nucleic Acids Res 22:5767–5768. 10.1093/nar/22.25.5767 7838736 10.1093/nar/22.25.5767 PMC310147

[CR33] Nai C, Wong HY, Pannenbecker A, Broughton WJ, Benoit I, de Vries RP et al (2013) Nutritional physiology of a rock-inhabiting, model microcolonial fungus from an ancestral lineage of the Chaetothyriales (Ascomycetes). Fungal Genet Biol 56:54–66. 10.1016/j.fgb.2013.04.001 23587800 10.1016/j.fgb.2013.04.001

[CR34] Noack-Schönmann S, Bus T, Banasiak R, Knabe N, Broughton WJ, Den Dulk-Ras H et al (2014) Genetic transformation of *Knufia petricola* A95 - a model organism for biofilm-material interactions. AMB Express 4:80. 10.1186/s13568-014-0080-5 25401079 10.1186/s13568-014-0080-5 PMC4230810

[CR35] Nødvig CS, Nielsen JB, Kogle ME, Mortensen UH (2015) A CRISPR-Cas9 system for genetic engineering of filamentous fungi. PLoS ONE 10:e0133085. 10.1371/journal.pone.0133085 26177455 10.1371/journal.pone.0133085 PMC4503723

[CR36] Nødvig CS, Hoof JB, Kogle ME, Jarczynska ZD, Lehmbeck J, Klitgaard DK, Mortensen UH (2018) Efficient oligo nucleotide mediated CRISPR-Cas9 gene editing in Aspergilli. Fungal Genet Biol 115:78–89. 10.1016/j.fgb.2018.01.004 29325827 10.1016/j.fgb.2018.01.004

[CR37] Oldenburg KR, Vo KT, Michaelis S, Paddon C (1997) Recombination-mediated PCR-directed plasmid construction in vivo in yeast. Nucleic Acids Res 25:451–452. 10.1093/nar/25.2.451 9016579 10.1093/nar/25.2.451 PMC146432

[CR38] Peng Y, Zhang H, Xu M, Tan MW (2018) A Tet-Off gene expression system for validation of antifungal drug targets in a murine invasive pulmonary aspergillosis model. Sci Rep 8:443. 10.1038/s41598-017-18868-9 29323188 10.1038/s41598-017-18868-9 PMC5765126

[CR39] Prenafeta-Boldú FX, Medina-Armijo C, Isola D (2022) Black fungi in the built environment-the good, the bad, and the ugly. Viruses, Bacteria and Fungi in the Built Environment. Woodhead Publishing, pp 65–99. 10.1016/B978-0-323-85206-7.00008-3

[CR40] Ruibal C, Selbmann L, Avci S, Martin-Sanchez PM, Gorbushina AA (2018) Roof-inhabiting cousins of rock-inhabiting fungi: novel melanized microcolonial fungal species from photocatalytically reactive subaerial surfaces. Life (Basel) 8:30. 10.3390/life8030030 30011950 10.3390/life8030030 PMC6161114

[CR41] Sandmann G (2022) Carotenoids and their biosynthesis in fungi. Molecules 27:1431. 10.3390/molecules27041431 35209220 10.3390/molecules27041431 PMC8879039

[CR42] Sasse A, Hamer SN, Amich J, Binder J, Krappmann S (2016) Mutant characterization and in vivo conditional repression identify aromatic amino acid biosynthesis to be essential for *Aspergillus fumigatus* virulence. Virulence 7:56–62. 10.1080/21505594.2015.1109766 26605426 10.1080/21505594.2015.1109766 PMC4871646

[CR43] Schuetze T, Meyer V (2017) Polycistronic gene expression in *Aspergillus niger*. Microb Cell Fact 16:162. 10.1186/s12934-017-0780-z 28946884 10.1186/s12934-017-0780-z PMC5613464

[CR44] Schumacher J (2012) Tools for *Botrytis cinerea*: new expression vectors make the gray mold fungus more accessible to cell biology approaches. Fungal Genet Biol 49:483–497. 10.1016/j.fgb.2012.03.005 22503771 10.1016/j.fgb.2012.03.005

[CR45] Schumacher J, Gorbushina AA (2020) Light sensing in plant- and rock-associated black fungi. Fungal Biol 124:407–417. 10.1016/j.funbio.2020.01.004 32389303 10.1016/j.funbio.2020.01.004

[CR46] Selbmann L, Zucconi L, Isola D, Onofri S (2015) Rock black fungi: excellence in the extremes, from the Antarctic to space. Curr Genet 61:335–345. 10.1007/s00294-014-0457-7 25381156 10.1007/s00294-014-0457-7

[CR47] Selbmann L, Benko Z, Coleine C, de Hoog S, Donati C, Druzhinina I et al (2020) Shed Light in the DaRk lineages of the fungal tree of life-STRES. Life (Basel) 10:362. 10.3390/life10120362 33352712 10.3390/life10120362 PMC7767062

[CR48] Staley JT, Palmer F, Adams JB (1982) Microcolonial fungi: common inhabitants on desert rocks? Science 215:1093–1095. 10.1126/science.215.4536.1093 17771840 10.1126/science.215.4536.1093

[CR49] Tesei D (2022) Black fungi research: out-of-this-world implications. Encyclopedia 2:212–229. 10.3390/encyclopedia2010013

[CR50] Tonon C, Breitenbach R, Voigt O, Turci F, Gorbushina AA, Favero-Longo SE (2021) Hyphal morphology and substrate porosity -rather than melanization- drive penetration of black fungi into carbonate substrates. J Cult Herit 48:244–253. 10.1016/j.culher.2020.11.003

[CR51] Ugolini S, Bruschi CV (1996) The red/white colony color assay in the yeast *Saccharomyces cerevisiae*: epistatic growth advantage of white ade8-18, ade2 cells over red ade2 cells. Curr Genet 30:485–492. 10.1007/s002940050160 8939809 10.1007/s002940050160

[CR52] Verwaal R, Wang J, Meijnen JP, Visser H, Sandmann G, van den Berg JA, van Ooyen AJ (2007) High-level production of beta-carotene in *Saccharomyces cerevisiae* by successive transformation with carotenogenic genes from *Xanthophyllomyces dendrorhous*. Appl Environ Microbiol 73:4342–4350. 10.1128/AEM.02759-06 17496128 10.1128/AEM.02759-06 PMC1932764

[CR53] Vogt K, Bhabhra R, Rhodes JC, Askew DS (2005) Doxycycline-regulated gene expression in the opportunistic fungal pathogen *Aspergillus fumigatus*. BMC Microbiol 5:1. 10.1186/1471-2180-5-1 15649330 10.1186/1471-2180-5-1 PMC546209

[CR54] Voigt O, Knabe N, Nitsche S, Erdmann EA, Schumacher J, Gorbushina AA (2020) An advanced genetic toolkit for exploring the biology of the rock-inhabiting black fungus Knufia petricol. Sci Rep 10:22021. 10.1038/s41598-020-79120-5 33328531 10.1038/s41598-020-79120-5 PMC7745021

[CR55] Volkmann M, Whitehead K, Rutters H, Rullkotter J, Gorbushina AA (2003) Mycosporine-glutamicol-glucoside: a natural UV-absorbing secondary metabolite of rock-inhabiting microcolonial fungi. Rapid Commun Mass Spectrom 17:897–902. 10.1002/rcm.997 12717761 10.1002/rcm.997

[CR56] Wanka F, Cairns T, Boecker S, Berens C, Happel A, Zheng X et al (2016) Tet-on, or Tet-off, that is the question: advanced conditional gene expression in Aspergillus. Fungal Genet Biol 89:72–83. 10.1016/j.fgb.2015.11.003 26555930 10.1016/j.fgb.2015.11.003

[CR57] Winston F, Dollard C, Ricupero-Hovasse SL (1995) Construction of a set of convenient *Saccharomyces cerevisiae* strains that are isogenic to S288C. Yeast 11:53–55. 10.1002/yea.320110107 7762301 10.1002/yea.320110107

[CR58] Wollenzien U (1995) On the isolation of microcolonial fungi occurring on and in marble and other calcareous rocks. Sci Total Environ 167:287–294. 10.1016/0048-9697(95)04589-S

[CR59] Wollenzien U, De Hoog G, Krumbein W, Uijthof J (1997) *Sarcinomyces petricola*, a new microcolonial fungus from marble in the Mediterranean basin. Antonie Van Leeuwenhoek 71:281–288. 10.1023/a:1000157803954 9111924 10.1023/a:1000157803954

[CR60] Zheng X, Cairns T, Zheng P, Meyer V, Sun J (2022) Protocol for gene characterization in *Aspergillus niger* using 5S rRNA-CRISPR-Cas9-mediated Tet-on inducible promoter exchange. STAR Protoc 3:101838. 10.1016/j.xpro.2022.101838 36595926 10.1016/j.xpro.2022.101838 PMC9678785

