# Supplementary material for: The Tet-on system for controllable gene expression in the rock-inhabiting black fungus Knufia petricola
Source: Extremophiles. 2024 Aug 6;28(3):38. doi: 10.1007/s00792-024-01354-2 (PMC11303440; doi:10.1007/s00792-024-01354-2)
Supplement: Supplementary file 1 — Supplementary file1 (PDF 1848 KB) [file 792_2024_1354_MOESM1_ESM.pdf]

Electronic Supplementary Material

The Tet-on system for controllable gene expression in the rock-inhabiting black fungus *Knufia petricola*

Eileen A. Erdmann [1,2], Antonia K. M. Brandhorst [1,2], Anna A. Gorbushina [1,2], and Julia Schumacher [1,2*]

[1] *Bundesanstalt für Materialforschung und -prüfung (BAM), Berlin, Germany*
[2] *Freie Universität Berlin, Berlin, Germany*

* Corresponding author: Julia.Schumacher@bam.de

Supplementary FIGURES 2

- Fig S1 Intergenic regions for neutral insertion of expression constructs in the *K. petricola* genome..... 2
- Fig S2 Cloning of TET-containing expression constructs for integration in *K. petricola igr1* or *igr2*. 3
- Fig S3 *Igr3*, *igr4*, and *igr5* are additional neutral insertion sites in the *K. petricola* genome. 4
- Fig S4 Dose-dependent expression of White collar-like transcription factors from neutral genomic loci..... 5
- Fig S5 Generation of *K. petricola* Δ*pks1*/Δ*phs1-phd1* mutants. 6
- Fig S6 Constructs for expressing the carotenogenic genes from *K. petricola* in different hosts..... 7
- Fig S7 Expression of the carotenogenic *K. petricola* genes from pIGRXR-TET in *S. cerevisiae*. 8

Supplementary TABLES 9

- Table S1 *K. petricola* strains used in this study. 9
- Table S2 GenBank accession numbers of studied *K. petricola* genomic loci..... 11
- Table S3 *S. cerevisiae* strains used in this study. 11
- Table S4 Oligonucleotides used for cloning and generation of donor DNA in this study..... 12
- Table S5 Oligonucleotides used for sequencing and diagnostic PCR in this study..... 16
- Table S6 Plasmids cloned in this study..... 18
- Table S7 Transformations of *K. petricola* protoplasts carried out in this study..... 24

Supplementary REFERENCES 26

Supplementary FIGURES

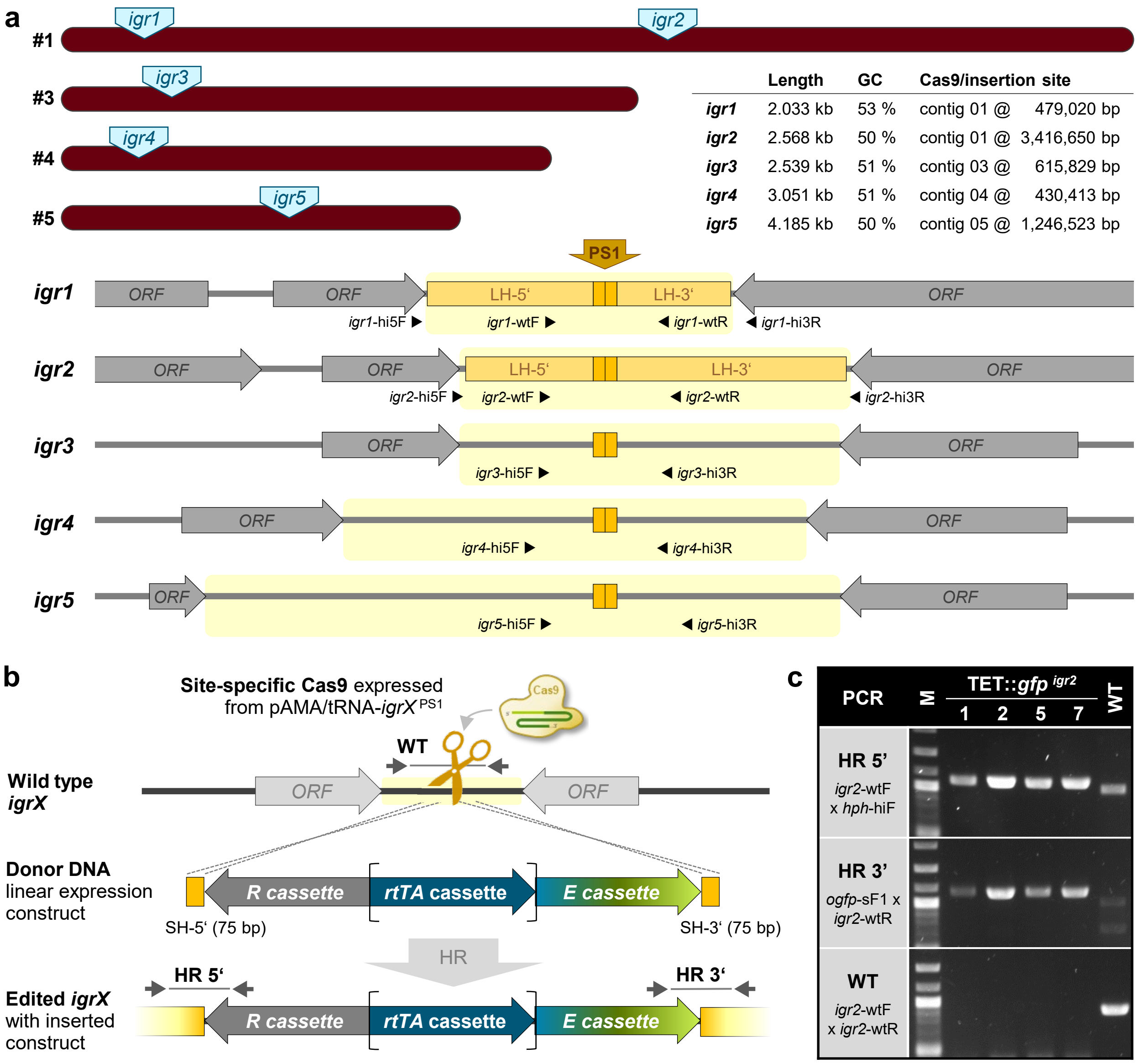

Fig S1 Intergenic regions for neutral insertion of expression constructs in the *K. petricola* genome.

(a) Five intergenic regions on four different contigs were chosen. *Igr1* and *igr2* have been validated previously (Erdmann et al. 2022). Three additional regions on other contigs of the *K. petricola* A95 genome assembly were chosen. Shown are the insertion sites (at Cas9-mediated DSBs via *igr*-specific PS1), homologous sequences contained in donor DNA (orange – short homologous (SH) sequences flanking PCR-generated donor DNA, light orange – long homologous (LH) sequences flanking constructs assembled in pIGR1/2R(-TGG) cloning vectors), and primers used for detection of targeted insertion by homologous recombination (HR). **(b) Strategies for targeted integration of expression constructs.** Cloning vectors containing LH sequences to *igr1* or *igr2* [pIGRXR(-XXX) series] can be used for the assembly of expression constructs for targeted integration. Expression constructs with SH sequences for the insertion in all *igr*s can be generated by PCR using appropriate primers and templates. Exemplarily shown is the insertion of expression constructs with SH sequences derived from pNXR-XXX cloning vectors, which carry the resistance (R) and expression (E) cassette in an inverse orientation. The *rtTA* cassette for the expression of the DOX-regulated transcription factor is present in TET constructs only. **(c) Identification of transformants carrying a TET::*gfp* construct at *igr2*.** The [(T*niaD*::*hph*::P*trpC*)–(P*oliC*::*rtTA*::T*crgA*)–(P*tet*::*gfp*::T*gluc*)] expression construct flanked by 75-bp-long homologous sequences to *igr2* was amplified from pNAH-OTGG using the primers T*niiA*-*igr2*-SH5F and T*gluc*-*igr2*-SH3R. Protoplasts of WT:A95 were transformed with the construct as donor DNA and the Cas9- and sgRNA-delivering pAMA/ribo-*igr2* PS1 (Table S7). By diagnostic PCR using primers binding to the ends of the R or E cassette and in *igr2*, four strains with correctly inserted expression constructs were identified.

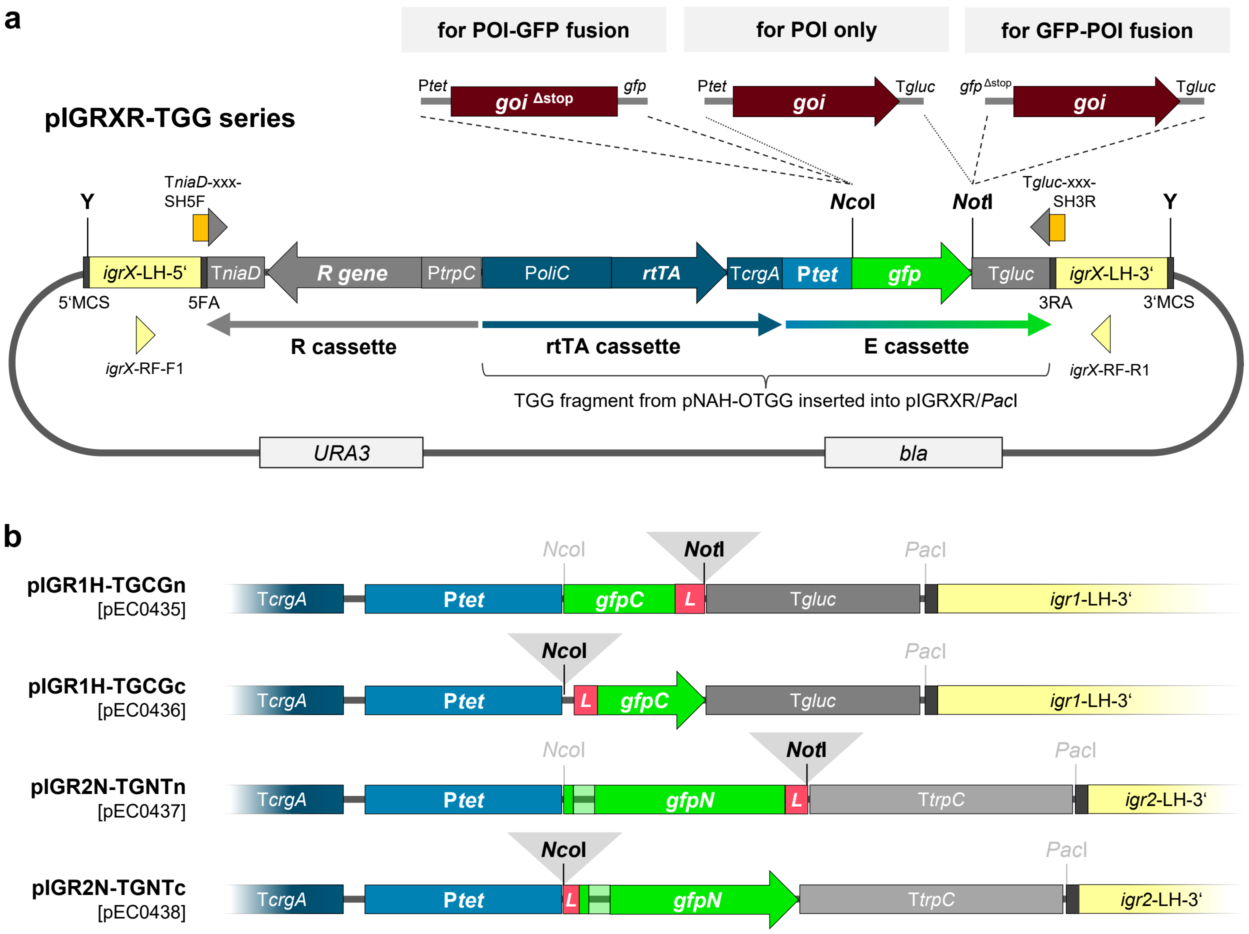

Fig S2 Cloning of TET-containing expression constructs for integration in *K. petricola igr1* or *igr2*.

(a) The pIGRXR-TGG series for TET-regulated expression of a *gene* or a *gene-gfp* fusion protein. Vectors were generated as follows: the TGG fragment shown was amplified by PCR from pNAH-OTGG (Janevska et al. 2017) with primers containing 5' overhangs to P*trpC* and the 3R-adapter, and assembled with *Pac*I-digested pIGRXR plasmids (Erdmann et al. 2022). In sum, twelve cloning vectors were cloned containing different resistance (R) genes flanked by *A. nidulans* P*trpC* and *B. cinerea* T*niaD*, and homologous sequences to *igr1* or *igr2* (Table S6). Genes of interest (*goi*) are inserted upstream of *gfp* for C-terminal GFP, downstream of *gfp* for N-terminal GFP or by replacing *gfp* using the restriction sites *Nco*I or *Not*I for linearization of the entry plasmid. Donor DNA for transformation of *K. petricola* i.e., linear expression constructs flanked by homologous sequences, can be generated by digestion of the plasmids with restriction enzymes cutting in the multiple cloning sites (MCS), by amplification with short primers *igrX*-RF-F1 and *igrX*-RF-R1, or by using primers binding to the terminators (T*niaD*-xxx-SH5F, T*gluc*-xxx-SH3R) and containing the 75-bp-long 5' overhangs for attaching SH sequences (shown as orange boxes). Further abbreviations: 5FA, 3RA – synthetic adapter sequences in the pIGRXR series; R gene – *hph*, *nat1*, *nptII*, *bar* and *sur* for mediating resistance against hygromycin B (HYG), nourseothricin (NTC), geneticin (G418), glufosinate (GFS) and chlorimuron ethyl (CME), respectively; POI – protein of interest. **(b) Vectors for cloning constructs for bimolecular fluorescence complementation (BiFC) studies.** The four vectors containing either a hygR cassette and the LH sequences of *igr1* or a natR cassette and the LH sequences of *igr2* were cloned using members of the pIGRXR-TGG series as entry plasmids (Table S6). *GfpC-linker* fragments were amplified from pNAH-OGCGn/c (Schumacher 2012) and assembled in *Nco*I+*Not*I-digested pIGR1H-TGG yielding pIGR1H-TGCGn and pIGR1H-TGCGc. For pIGR2N-TGNTn and pIGR2N-TGNTc, the *gfpN*-linker fragments were amplified with T*trpC* from pNDN-AGNTn/c (Schumacher 2012) and assembled in *Nco*I+*Pac*I-digested pIGR2N-TGG. Note that the linker (L) sequences differ, and that the used *gfp* sequence contains an intron in its 5' region (light green box). The sites for insertion of a *goi* – with stop codon for N-terminal GFP-Linker (n) and without stop codon for C-terminal Linker-GFP (c) – are indicated by gray arrows.

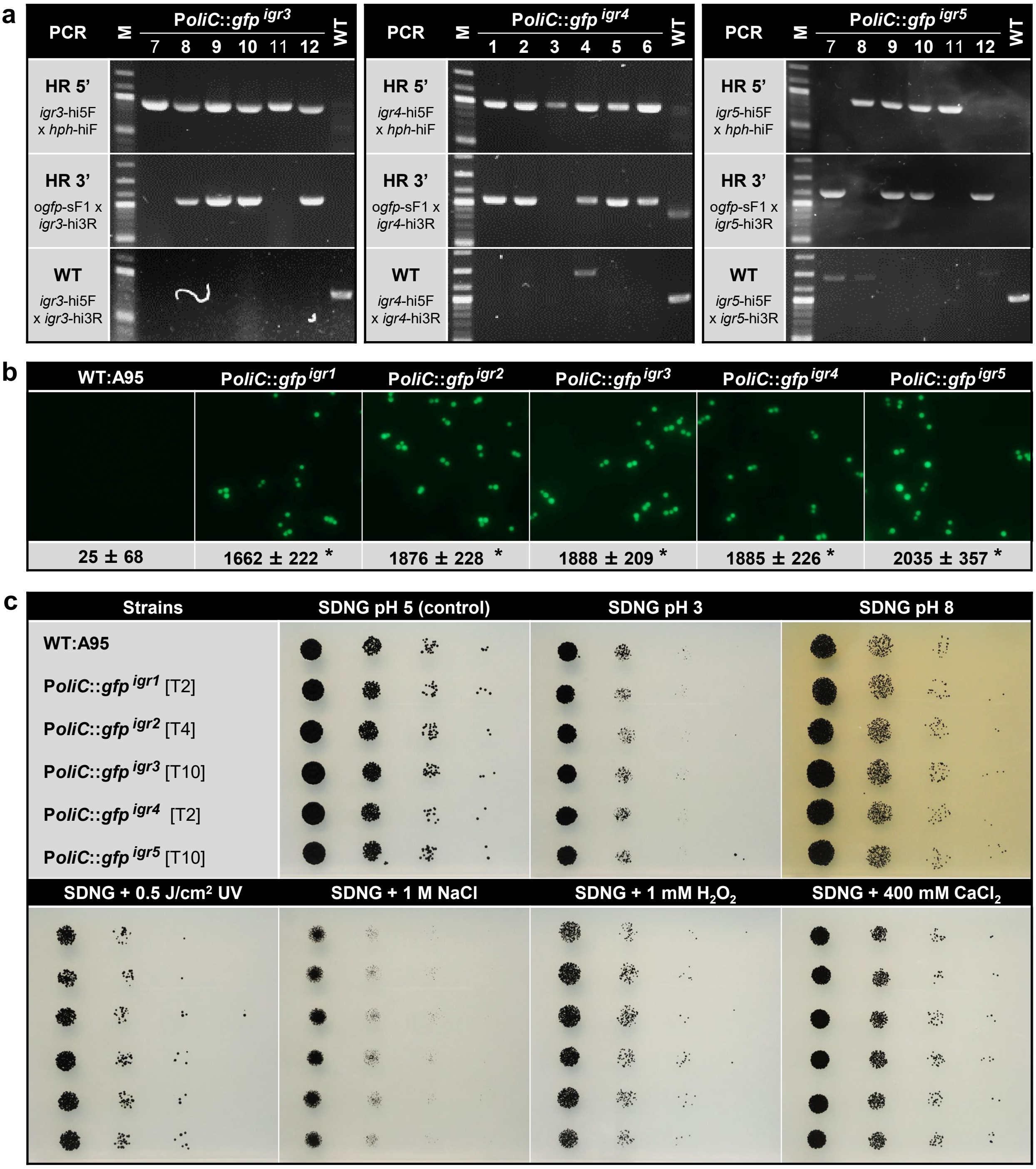

Fig S3 *Igr3*, *igr4*, and *igr5* are additional neutral insertion sites in the *K. petricola* genome.

Strains with insertions at *igr3*, *igr4* or *igr5* were generated by transformation of WT:A95 protoplasts with pAMA/tRNA-*igrX*^{PS1} and [(T*niaD*::*hph*::P*trpC*)–(P*oliC*::*gfp*::T*gluc*)] expression constructs flanked by 75-bp-long homologous sequences to *igr*X. Strains containing the same expression constructs in *igr1* and *igr2* were available from a previous study. **(a) Strains with targeted integration of the GFP expression constructs were identified by diagnostic PCRs.** Combinations of primers binding in the *igr*X or the construct (Fig S1) were used for detecting HR events and the absence of the wild type locus. **(b) GFP is equally well expressed from all five intergenic regions.** Cells of two-day-old liquid cultures were dropped onto objective slides and submitted to fluorescence microscopy. Images were captured with exposure times of 40 ms. Mean values and standard deviations of GFP fluorescence intensities derived from 100 cells of two transformants per strain and 50 cells of WT. * $p < 0.001$ compared to WT. **(c) Insertions in the intergenic regions do not affect growth under different stress conditions.** Cells (10^4, 10^3, 10^2, 10^1) were dropped onto solid SDNG pH 5 (control) and modified media as shown. Pictures were taken 10 dpi.

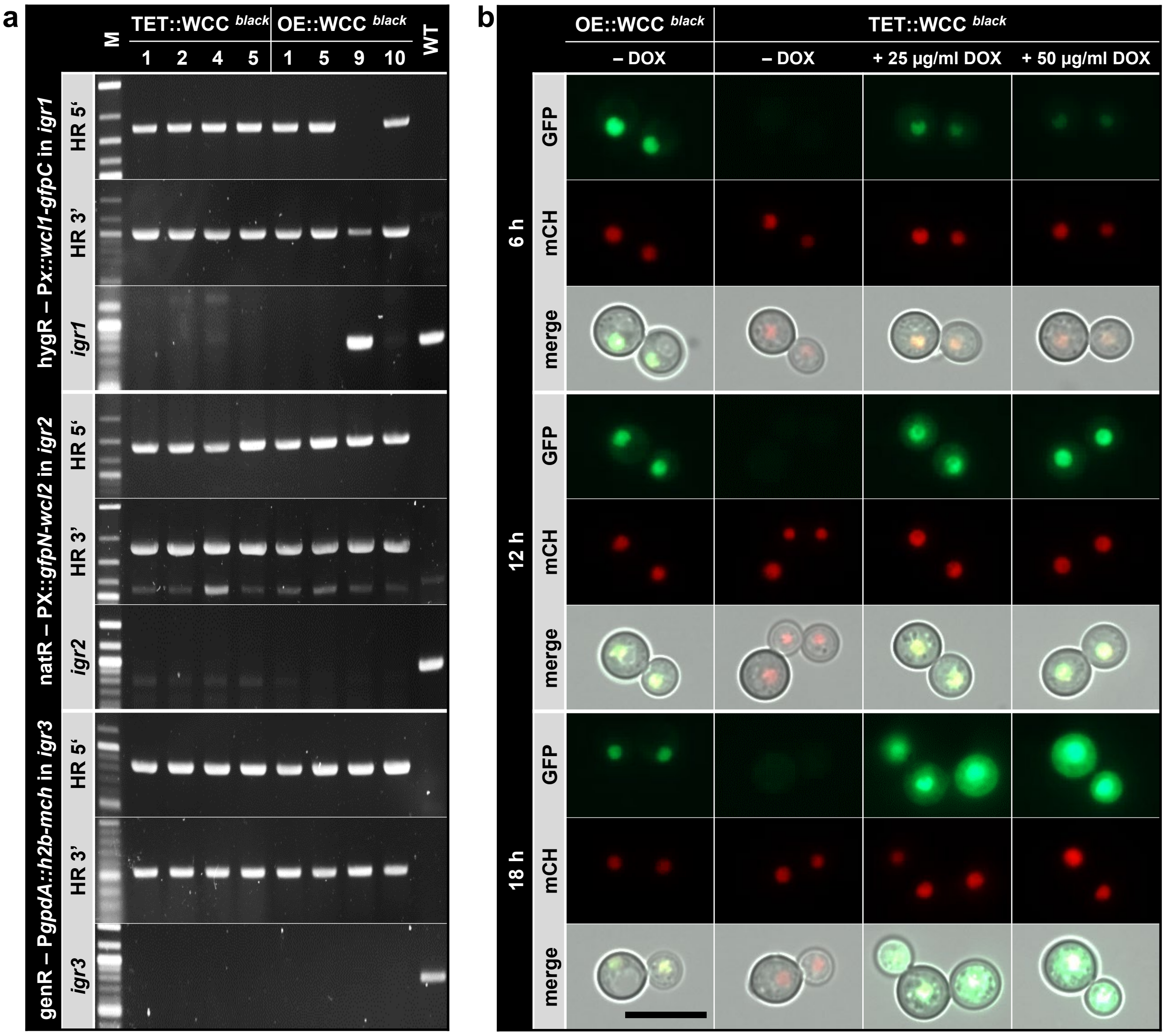

Fig S4 Dose-dependent expression of White collar-like transcription factors from neutral genomic loci.

(a) Verification of the integration of the three constructs in *igr1*, *igr2*, and *igr3*, respectively. Four HYG-, NTC- and G418-resistant transformants of OE::WCC *black* and TET::WCC *black* strains were screened by diagnostic PCR using primers binding to the ends of the constructs and in *igrX*. Amplicons for HR and the absence of the wild type intergenic regions verified the correct insertions. **(b) Identification of optimal DOX concentration and induction time for protein-protein interaction studies.** Two-day-old liquid cultures of OE::WCC *black* (T1) and TET::WCC *black* (T2) were split in five one ml cultures for incubation without and with 25, 50, 75 and 100 µg/ml DOX. 100 µl of the cultures were sampled at the indicated time points and directly used for fluorescence microscopy with exposure times of 80 ms (mCH) and 800 ms (GFP). Scale bar – 10 µm.

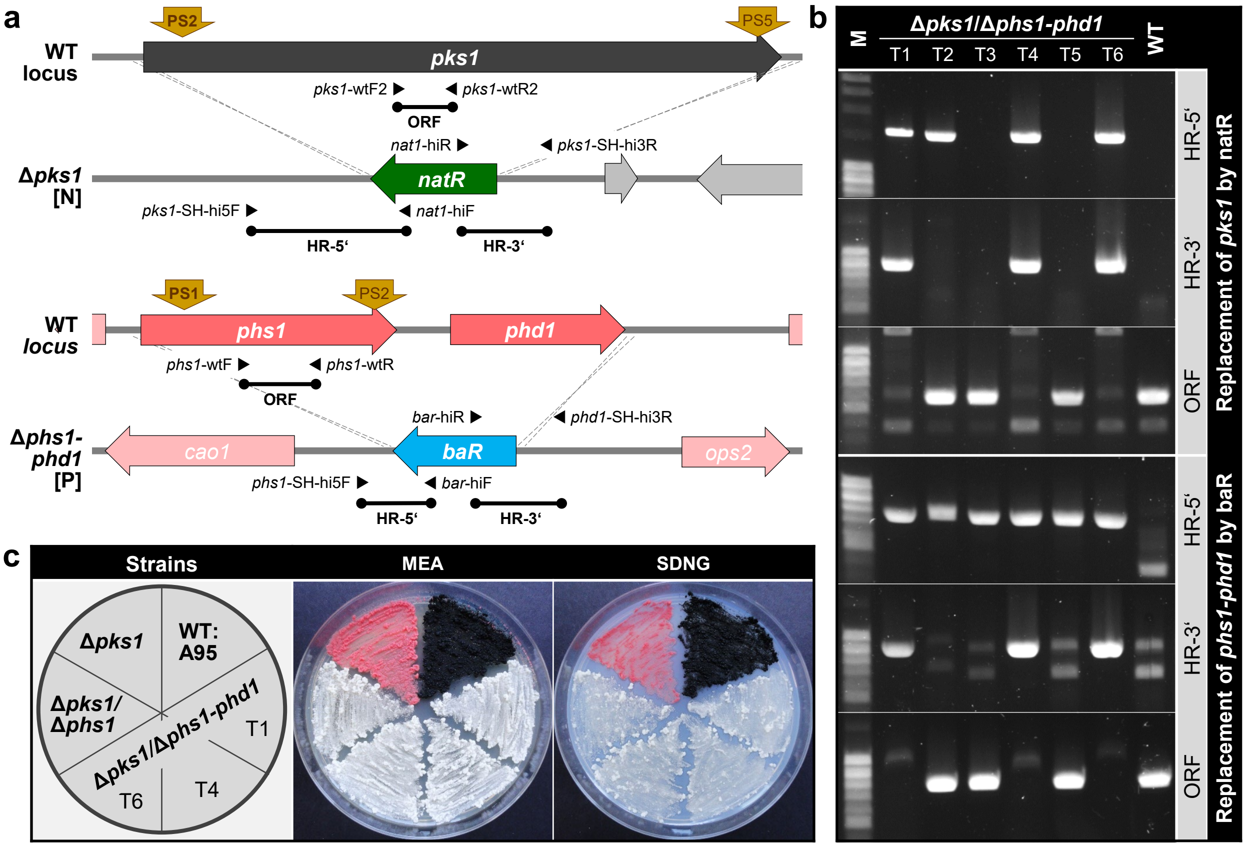

Fig S5 Generation of *K. petricola* Δ*pks1*/Δ*phs1-phd1* mutants.

(a) Strategies for simultaneous replacement of *pks1*, *phs1* and *phd1*. Protoplasts of WT:A95 were co-transformed with the Cas9- and sgRNA delivering plasmid pAMA/tRNA-*pks1*^{PS2}-*phs1*^{PS1} and resistance cassette-containing donor DNA with 75-bp-long homologous sequences to the non-coding regions up- and downstream of *pks1* or *phs1-phd1* (Table S7). *Pks1* was replaced by a natR cassette, the physically linked *phs1* and *phd1* by a glufosinate resistance (baR) cassette. The location of Cas9 sites (protospacers, PS) for introduction of DSBs in the coding regions of *pks1* and *phs1* are shown. **(b) Diagnostic PCRs confirmed the replacement of *pks1* and physically linked *phs1* and *phd1* in three transformants.** Six resistant transformants were screened by PCR for HR events and the absence of the genes of interest (ORF). Primers binding up- or downstream of genes of interest or to the resistance cassettes were used as shown in a. Transformants T1, T4, and T6 exhibit the expected amplicon pattern. **(c) The three mutants exhibit an albino phenotype.** Wild type A95, the newly generated mutants and available mutants defective in melanogenesis (Δ*pks1*) or melanogenesis and carotenogenesis (Δ*pks1*/Δ*phs1*) for comparison were cultivated for ten days on solidified MEA and SDNG.

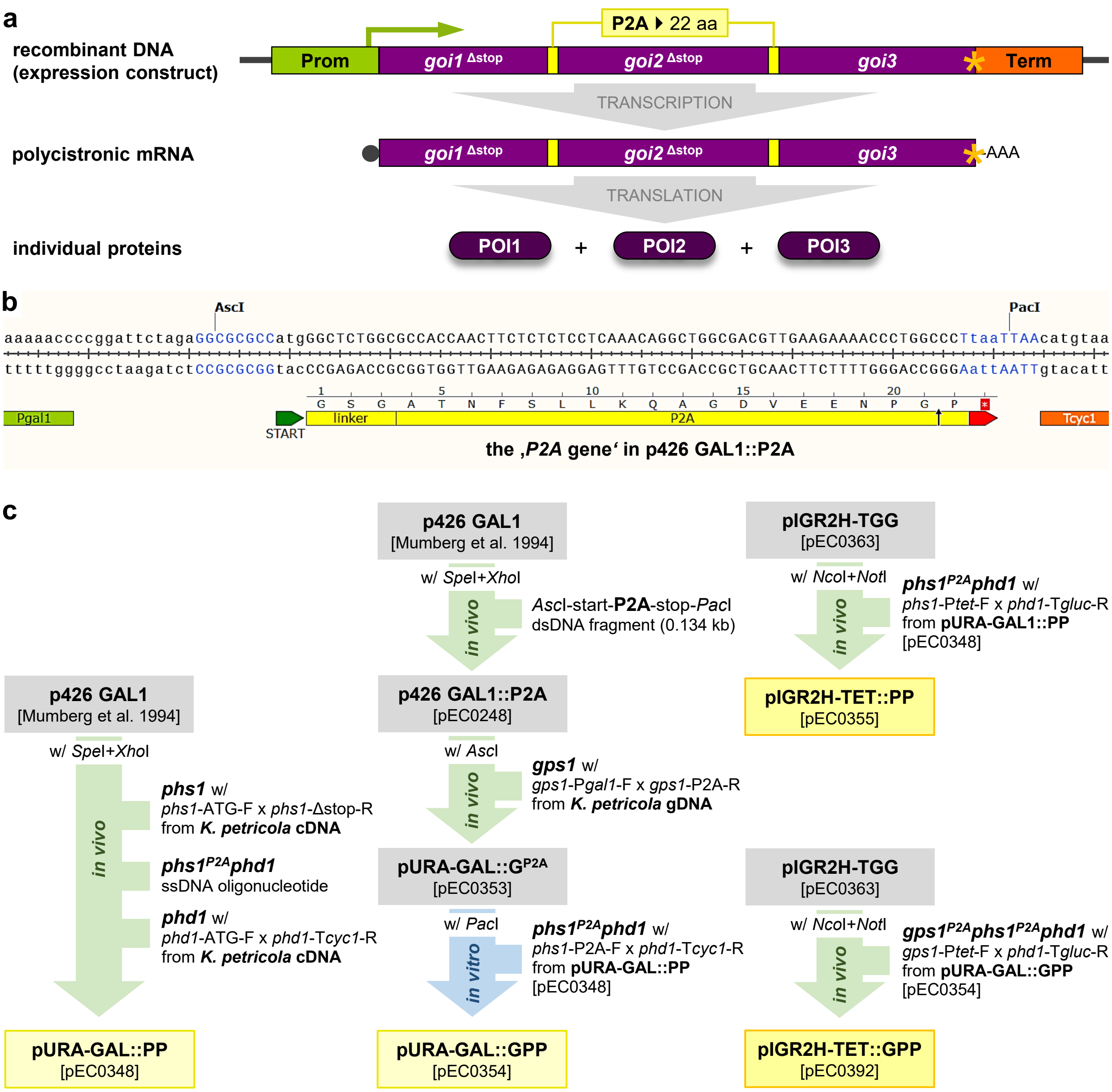

Fig S6 Constructs for expressing the carotenogenic genes from *K. petricola* in different hosts.

(a) Strategy for expressing two or more proteins from a polycistronic mRNA. P2A peptides can induce ribosomal skipping during translation of a protein in a cell. They share the core sequence motif DxExNPGP and are found in a wide range of viral families. They help generating polyproteins by causing the ribosome to fail at making a peptide bond. **(b) A synthetic P2A-encoding gene was inserted into p426 GAL1 for inserting *goi* for expression in yeast.** A GSG spacer was included to improve the cleavage efficiency of P2A peptides. The black arrow represents the cleavage site. After self-cleavage, glycine is the last amino acid of the upstream protein, while proline is the first amino acid of the downstream protein. Restriction sites for the rarely cutting enzymes *Asc*I and *Pac*I (sequences in blue) and start (ATG) and stop codons (TAA) were inserted up- and downstream of the GSG-P2A sequences. One or more genes can be inserted. This entry plasmid also allows for testing the effect of the attached P2A on the activity of a single protein by inserting the gene upstream of the P2A motif. p426 GAL1 is an *E. coli*/*S. cerevisiae* shuttle vector containing the full-length *S. cerevisiae GAL1* promoter and *CYC1* terminator which flank a cloning site for insertion of a *goi*, and *URA3* and the 2-micron for selection and propagation in *S. cerevisiae* (Mumberg et al. 1994). **(c) The four plasmids for the expression of two or three carotenogenic genes were cloned in several steps.** Cloning involved the generation of several intermediates and was accomplished by the digestion of an entry plasmid (in gray boxes) and its assembly with amplicons (primers and templates are shown), single-stranded (ss) or double-stranded (ds) synthetic DNA (see Table S6). *K. petricola* genes were amplified in the first step from cDNA to obtain intron-free genes for the expression in *S. cerevisiae*. The DNA assembly was performed in *S. cerevisiae* (*in vivo*) or *in vitro*.

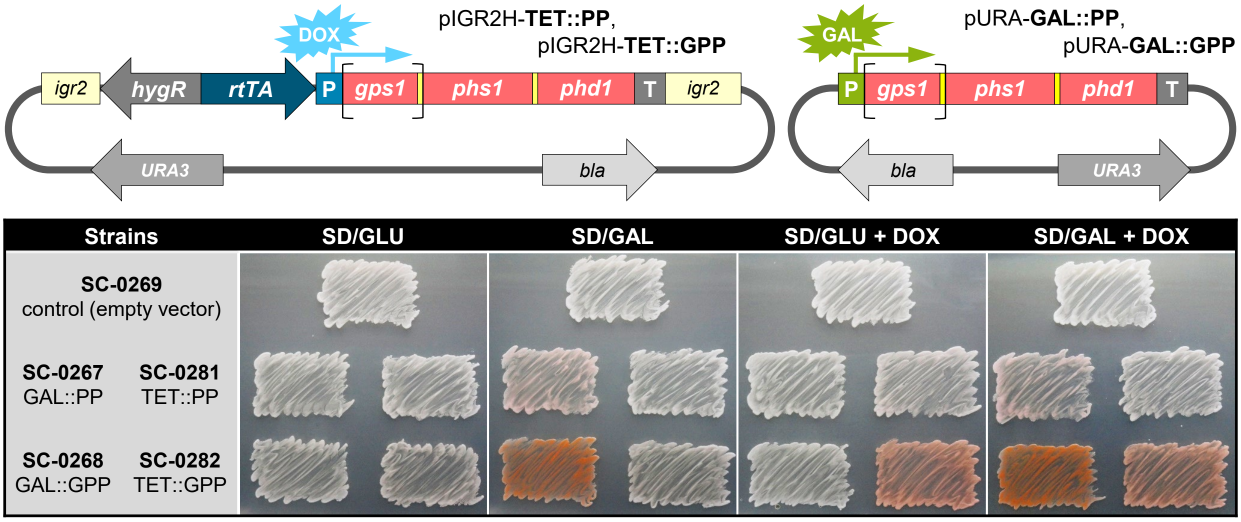

Fig S7 Expression of the carotenogenic *K. petricola* genes from pIGRXR-TET in *S. cerevisiae*.

pIGR2H-TET::(G)PP containing the intron-free variants of the carotenogenic *K. petricola* genes carry the 2-micron and *S. cerevisiae URA3* in their backbones. Consequently, they may mediate episomal gene expression in *S. cerevisiae*, as far as the synthetic transactivator-encoding gene is expressed. For control, yeast expression vectors were cloned (pURA-GAL::(G)PP) that contained the fused carotenogenic genes downstream of the *S. cerevisiae GAL1* promoter for galactose-inducible gene expression (Fig. S6c). The plasmid-carrying *S. cerevisiae* strains were cultivated for three days on selective medium (SD-URA) containing 2 % glucose (GLU) or 4 % galactose (GAL) and/or 10 µg/ml DOX.

Supplementary TABLES

Table S1 *K. petricola* strains used in this study.

Strain name	GMO ID	Clone #	Genotype	Reference
**WT**	n/a	n/a	A95	Nai et al. (2013)
Δpks1	**KP-0037**	PH1	A95, ***Δpks1*** [(T*niaD*::*hph*::P*trpC*)]	Voigt et al. (2020)
Δpks1	**KP-0033**	PN2	A95, ***Δpks1*** [(T*niaD*::*nat1*::P*trpC*)]	Voigt et al. (2020)
Δpks1/Δphs1	**KP-0083**	T4, 5	A95, ***Δpks1*** [(T*niaD*::*nat1*::P*trpC*)], ***Δphs1*** [(T*niaD*::*hph*::P*trpC*)]	Voigt et al. (2020)
**TET::*gfp*** ^{igr1}	**KP-0460**	T1, 4	A95, ***igr1*** [(T*niaD*::*hph*::P*trpC*)–(P*oliC*::*rtTA*::T*crgA*)–(P*tet*::***gfp***::T*gluc*)]	This study
**TET::*gfp*** ^{igr2}	**KP-0256**	T1, 2, 5, 7	A95, ***igr2*** [(T*niaD*::*hph*::P*trpC*)–(P*oliC*::*rtTA*::T*crgA*)–(P*tet*::***gfp***::T*gluc*)]	This study
**P*gpdA*::*gfp*** ^{igr1}	**KP-0172**	T1, 2	A95, ***igr1*** [(T*niaD*::*hph*::P*trpC*)–(P*gpdA*::***gfp***::T*gluc*)]	Erdmann et al. (2022)
**P*gpdA*::*gfp*** ^{igr2}	**KP-0176**	T1, 4	A95, ***igr2*** [(T*niaD*::*hph*::P*trpC*)–(P*gpdA*::***gfp***::T*gluc*)]	Erdmann et al. (2022)
**P*oliC*::*gfp*** ^{igr1}	**KP-0173**	T1, 2	A95, ***igr1*** [(T*niaD*::*hph*::P*trpC*)–(P*oliC*::***gfp***::T*gluc*)]	Erdmann et al. (2022)
**P*oliC*::*gfp*** ^{igr2}	**KP-0177**	T2, 4	A95, ***igr2*** [(T*niaD*::*hph*::P*trpC*)–(P*oliC*::***gfp***::T*gluc*)]	Erdmann et al. (2022)
Δade2	**KP-0055**	T2	A95, ***Δade2*** [(T*niaD*::*nat1*::P*trpC*)]	Voigt et al. (2020)
Δpks1/Δphs1/Δade2	**KP-0282**	T2, 6, 16	A95, ***Δpks1*** [(T*niaD*::*nat1*::P*trpC*)], ***Δphs1*** [(T*niaD*::*hph*::P*trpC*)], ***Δade2*** [(T*niaD*::*bar*::P*trpC*)]	This study
**TET::WCC** ^{black}	**KP-0502**	T1, 2, 4, 5	A95, ***igr1*** [(T*niaD*::*hph*::P*trpC*)–(P*oliC*::*rtTA*::T*crgA*)–(P*tet*::***wcl1-gfpC***::T*gluc*)],<br>***igr2*** [(T*niaD*::*nat1*::P*trpC*)–(P*oliC*::*rtTA*::T*crgA*)–(P*gpdA*::***gfpN-wcl2***::T*trpC*)],<br>***igr3*** [(T*niaD*::*nptII*::P*trpC*)–(P*gpdA*::***smh2b-mch***::T*trpC*)]	This study
**OE::WCC** ^{black}	**KP-0503**	T1, 5,10	A95, ***igr1*** [(T*niaD*::*hph*::P*trpC*)–(P*gpdA*::***wcl1-gfpC***::T*gluc*)],<br>***igr2*** [(T*niaD*::*nat1*::P*trpC*)–(P*gpdA*::***gfpN-wcl2***::T*trpC*)],<br>***igr3*** [(T*niaD*::*nptII*::P*trpC*)–(P*gpdA*::***smh2b-mch***::T*trpC*)]	This study
**TET::WCC** ^{rose}	**KP-0484**	T5, 9, 17	A95, ***Δpks1*** [(T*niaD*::*hph*::P*trpC*)–(P*oliC*::*rtTA*::T*crgA*)–(P*tet*::***wcl1-gfpC***::T*gluc*)],<br>***Δphs1*** [(T*niaD*::*nat1*::P*trpC*)–(P*oliC*::*rtTA*::T*crgA*)–(P*gpdA*::***gfpN-wcl2***::T*trpC*)],<br>***Δade2*** [(T*niaD*::*nptII*::P*trpC*)–(P*gpdA*::***smh2b-mch***::T*trpC*)]	This study
**OE::WCC** ^{rose}	**KP-0485**	T4, 6, 12	A95, ***Δpks1*** [(T*niaD*::*hph*::P*trpC*)–(P*gpdA*::***wcl1-gfpC***::T*gluc*)],<br>***Δphs1*** [(T*niaD*::*nat1*::P*trpC*)–(P*gpdA*::***gfpN-wcl2***::T*trpC*)],<br>***Δade2*** [(T*niaD*::*nptII*::P*trpC*)–(P*gpdA*::***smh2b-mch***::T*trpC*)]	This study
**P*oliC*::*gfp*** ^{igr3}	**KP-0426**	T5, 8, 9, 10	A95, ***igr3*** [(T*niaD*::*hph*::P*trpC*)–(P*oliC*::***gfp***::T*gluc*)]	This study

Strain name	GMO ID	Clone #	Genotype	Reference
PoliC*::*gfp* *igr4	**KP-0427**	T1, 2, 5, 6	A95, ***igr4*** [(T*niaD*::*hph*::P*trpC*)–(P*oliC*::***gfp***::T*gluc*)]	This study
PoliC*::*gfp* *igr5	**KP-0428**	T9, 10	A95, ***igr5*** [(T*niaD*::*hph*::P*trpC*)–(P*oliC*::***gfp***::T*gluc*)]	This study
‚orange'	**KP-0201**	O1	A95; ***pks1–*** (Δ18 bp @ ~407 bp); ***phs1–*** (Δ9 bp @ ~345 bp)	Erdmann et al. (2022)
**Δ*pks1*/Δ*phs1-phd1***	**KP-0289**	T1, 4, 6	A95, **Δ*pks1*** [(T*niaD*::*nat1*::P*trpC*)], **Δ*phs1-phd1*** [(T*niaD*::*bar*::P*trpC*)]	This study
**TET::*pp***	**KP-0374**	T1, 2, 3	A95, **Δ*pks1*** [(T*niaD*::*nat1*::P*trpC*)], **Δ*phs1-phd1*** [(T*niaD*::*bar*::P*trpC*)],<br>***igr2*** [(T*niaD*::*hph*::P*trpC*)–(P*oliC*::*rtTA*::T*crgA*)–(P*tet*::***phs1-2A-phd1***::T*gluc*)]	This study
**TET::*gpp***	**KP-0383**	T1, 3, 6	A95, **Δ*pks1*** [(T*niaD*::*nat1*::P*trpC*)], **Δ*phs1-phd1*** [(T*niaD*::*bar*::P*trpC*)],<br>***igr2*** [(T*niaD*::*hph*::P*trpC*)–(P*oliC*::*rtTA*::T*crgA*)–(P*tet*::***gps1-2A-phs1-2A-phd1***::T*gluc*)]	This study
**TET::*pks1***	**KP-0454**	T1, 2, 3	A95, Δ***pks1*** [(T*niaD*::*nat1*::P*trpC*)], Δ***phs1-phd1*** [(T*niaD*::*bar*::P*trpC*)],<br>***igr2*** [(T*niaD*::*hph*::P*trpC*)–(P*oliC*::*rtTA*::T*crgA*)–(P*tet*::***pks1***::T*gluc*)]	This study
**Δ*ura3***	**KP-0032**	D2	A95, **Δ*ura3*** [(T*niaD*::*nat1*::P*trpC*)]	Voigt et al. (2020)
**Δ*ura3*/TET::*ura3* *igr1***	**KP-0459**	T4	A95, **Δ*ura3*** [(T*niaD*::*nat1*::P*trpC*)],<br>***igr1*** [(T*niaD*::*hph*::P*trpC*)–(P*oliC*::*rtTA*::T*crgA*)–(P*tet*::***ura3***::T*gluc*)]	This study
**Δ*ura3*/TET::*ura3* *igr2***	**KP-0415**	T2, 3, 4, 6	A95, **Δ*ura3*** [(T*niaD*::*nat1*::P*trpC*)],<br>***igr2*** [(T*niaD*::*hph*::P*trpC*)–(P*oliC*::*rtTA*::T*crgA*)–(P*tet*::***ura3***::T*gluc*)]	This study

Table S2 GenBank accession numbers of studied *K. petricola* genomic loci.

Name	Function	Nucleotide	Protein	Reference
**ADE2**	Phosphoribosylaminoimidazole carboxylase	**MT859423.1**	QOE76786.1	Voigt et al. (2020)
**URA3**	Orotidine 5'-phosphate decarboxylase	**MT859422.1**	QOE76785.1	Voigt et al. (2020)
**PKS1**	Polyketide synthase	**MT859418.1**	QOE76777.1	Voigt et al. (2020)
**GPS1**	Geranylgeranyl pyrophosphate synthase	**PP340520.1**	WWA97543.1	This study
**PHS1**	Lycopene cyclase/phytoene synthase	**MT859420.1**	QOE76780.1	Voigt et al. (2020)
**PHD1**	Phytoene desaturase	**MT859420.1**	QOE76781.1	Voigt et al. (2020)
**WCL1**	GATA-type transcription factor, white collar-like 1	**OM802157.1**	WAK13571.1	Erdmann et al. (2022)
**WCL2**	GATA-type transcription factor, white collar-like 2	**OM802158.1**	WAK13572.1	Erdmann et al. (2022)
igr1	Intergenic region 1	**OM802159.1**	n/a	Erdmann et al. (2022)
igr2	Intergenic region 2	**OM802160.1**	n/a	Erdmann et al. (2022)
igr3	Intergenic region 3	**PP340517.1**	n/a	This study
igr4	Intergenic region 4	**PP340518.1**	n/a	This study
igr5	Intergenic region 5	**PP340519.1**	n/a	This study

Table S3 *S. cerevisiae* strains used in this study.

Name	Expression construct	Strain, plasmid (auxotrophic marker)	Reference
**FY834**	n/a	Recipient	Winston et al. (1995)
**SC-0269**	**P*gal1*::*mcs*::T*cyc1***	FY834, p426 GAL1 (*URA3*)	This study
**SC-0267**	**P*gal1*::*phs1*^{2A}*phd1*::T*cyc1***	FY834, pURA-GAL::PP [pEC0348] (*URA3*)	This study
**SC-0268**	**P*gal1*::*gps1*^{2A}*phs1*^{2A}*phd1*::T*cyc1***	FY834, pURA-GAL::GPP [pEC0354] (*URA3*)	This study
**SC-0281**	**P*tet*::*phs1*^{2A}*phd1*::T*gluc***	FY834, pIGR2H-TET::PP [pEC0355] (*URA3*)	This study
**SC-0282**	**P*tet*::*gps1*^{2A}*phs1*^{2A}*phd1*::T*gluc***	FY834, pIGR2H-TET::GPP [pEC0392] (*URA3*)	This study

Genotype of FY834 is *MATα his3-Δ200 ura3-52 leu2-Δ1 lys2-Δ202 trp1-Δ63*.

Table S4 Oligonucleotides used for cloning and generation of donor DNA in this study.

Name	Sequence (5'→ 3')	Features (5'→ 3')
**pFC334-F1**	GGTCATAGCTGTTTCCGCTGA	MCS in pFC332/pFC902
**pFC334-R1**	TGATTCTGCTGTCTCGGCTG	P*tef1* in pFC332/pFC902
***Kppks1*-tRNA-PS2F**	tctgtgcaccggcttaataa-GTTTTAGAGCTAGAAATAGCAAGTTAAAAT	*kppks1*-PS2 – sgRNA
***Kppks1*-tRNA-PS2R**	ttattaagccggtgcacaga-TGCATCATCCGTGAATCGAAC	*kppks1*-PS2 – tRNA
***Kppks1*-tRNA-PS5R**	tcctacacttatgatctggg-TGCATCATCCGTGAATCGAAC	*kppks1*-PS5 – tRNA
***Kppks1*-tRNA-PS5F**	cccagatcataagtgtagga-GTTTTAGAGCTAGAAATAGCAAGTTAAAAT	*kppks1*-PS5 – gRNA
***Kpphs1*-tRNA-PS1F**	cggtgcagcttatccgcctg-GTTTTAGAGCTAGAAATAGCAAGTTAAAAT	*kpphs1*-PS1 – sgRNA
***Kpphs1*-tRNA-PS1R**	caggcggataagctgcaccg-TGCATCATCCGTGAATCGAAC	*kpphs1*-PS1 – tRNA
***Kpphs1*-tRNA-PS2R**	cgggcatagctaggtaaccc-TGCATCATCCGTGAATCGAAC	*kpphs1*-PS2 – tRNA
***Kpphs1*-tRNA-PS2F**	gggttacctagctatgcccg-GTTTTAGAGCTAGAAATAGCAAGTTAAAAT	*kpphs1*-PS2 – gRNA
***Kpade2*-tRNA-PS3R**	acgtcgacgacttcgagcaa-TGCATCATCCGTGAATCGAAC	*kpade2*-PS3 – tRNA
***Kpade2*-tRNA-PS3F**	ttgctcgaagtcgtcgacgt-GTTTTAGAGCTAGAAATAGCAAGTTAAAAT	*kpade2*-PS3 – gRNA
***Kpade2*-tRNA-PS2R**	cggccatggactctcatact-TGCATCATCCGTGAATCGAAC	*kpade2*-PS2 – tRNA
***Kpade2*-tRNA-PS2F**	agtatgagagtccatggccg-GTTTTAGAGCTAGAAATAGCAAGTTAAAAT	*kpade2*-PS2 – gRNA
***Kpade2*-tRNA-PS5R**	aggccgcgcggttggaggag-TGCATCATCCGTGAATCGAAC	*kpade2*-PS5 – tRNA
***Kpade2*-tRNA-PS5F**	ctcctccaaccgcgcggcct-GTTTTAGAGCTAGAAATAGCAAGTTAAAAT	*kpade2*-PS5 – gRNA
***Kpigr1*-tRNA-PS1F**	accgtactctccgcgtagac-GTTTTAGAGCTAGAAATAGCAAGTTAAAAT	*kpigr1*-PS1 – sgRNA
***Kpigr1*-tRNA-PS1R**	gtctacgcggagagtacggt-TGCATCATCCGTGAATCGAAC	*kpigr1*-PS1 – tRNA
***Kpigr2*-tRNA-PS1F**	agctgctagtccataactca-GTTTTAGAGCTAGAAATAGCAAGTTAAAAT	*kpigr2*-PS1 – sgRNA
***Kpigr2*-tRNA-PS1R**	tgagttatggactagcagct-TGCATCATCCGTGAATCGAAC	*kpigr2*-PS1 – tRNA
***Kpigr3*-tRNA-PS1F**	taagtcacgtgcactctaac-GTTTTAGAGCTAGAAATAGCAAGTTAAAAT	*kpigr3*-PS1 – sgRNA
***Kpigr3*-tRNA-PS1R**	gttagagtgcacgtgactta-TGCATCATCCGTGAATCGAAC	*kpigr3*-PS1 – tRNA
***Kpigr4*-tRNA-PS1F**	tccgcgcaaacctgggacgt-GTTTTAGAGCTAGAAATAGCAAGTTAAAAT	*kpigr4*-PS1 – sgRNA
***Kpigr4*-tRNA-PS1R**	acgtcccaggtttgcgcgga-TGCATCATCCGTGAATCGAAC	*kpigr4*-PS1 – tRNA
***Kpigr5*-tRNA-PS1F**	cattaccgttcgtcgggcgc-GTTTTAGAGCTAGAAATAGCAAGTTAAAAT	*kpigr5*-PS1 – sgRNA
***Kpigr5*-tRNA-PS1R**	gcgcccgacgaacggtaatg-TGCATCATCCGTGAATCGAAC	*kpigr5*-PS1 – tRNA
**P*trpC*-sF2**	GCTTGGTGCACGATAACTTGGTG	P*trpC* in R cassettes
***NptII*-mut-F1**	GATCTCGTCGTGACCCAcGGCGATGCCTGCTTGCCG	*nptII* $^{\Delta NcoI}$ (silent mutation)
***NptII*-mut-R1**	CGGCAAGCAGGCATCGCCgTGGGTCACGACGAGATC	*nptII* $^{\Delta NcoI}$ (silent mutation)

Name	Sequence (5'→ 3')	Features (5'→ 3')
***Sur*-mut-F1**	CACAAATGTCATCACTCCtATGGCCGACGCTCTTG	*sur* $^{\Delta NcoI}$ (silent mutation)
***Sur*-mut-R1**	CAAGAGCGTCGGCCATaGGAGTGATGACATTTGTG	*sur* $^{\Delta NcoI}$ (silent mutation)
***Sur*-mut-F2**	CTGGCGGTTTGGGAACaATGGGATATGGTCTGCCG	*sur* $^{\Delta NcoI}$ (silent mutation)
***Sur*-mut-R2**	CGGCAGACCATATCCCATtGTTCCCAAACCGCCAG	*sur* $^{\Delta NcoI}$ (silent mutation)
***Sur*-mut-F3**	GAAGGTTCCTGTTCTGCCgATGGTGCCCGGAGGTAAC	*sur* $^{\Delta NcoI}$ (silent mutation)
***Sur*-mut-R3**	GTTACCTCCGGGCACCATcGGCAGAACAGGAACCTTC	*sur* $^{\Delta NcoI}$ (silent mutation)
**T*niaD*-sR1**	GACTCACTGATACATCTGGCACCTAC	T*niaD* in R cassettes
**P*trpC*-3RAM-R2**	ggtggtgttcccgaatggaattggaatacc-ttaattaa-GATATTGAAGGAGCATTTTTTGGGCTTG	3R-adapter – *Pac*I – P*trpC*
**T*niaD*-5FAM-R**	ggagcgacgtcttgttcgcgatcttaagac-ggcgcgcc-GCATTGGATTAATAATTGTTGCTAAGCGAG	5F-adapter – *Asc*I – T*niaD*
***Kpigr1*-5F**	gttgggtaacgccagggttttcccagtcacga-atttaaatgggcccgtttaaacgctagc-CCGCCAGGTTGAACTCATGCTTAC	pRS426 – MCS – *kpigr1*
***Kpigr1*-5R**	gtcttaagatcgcgaacaagacgtcgctcc-TACGCGGAGAGTACGGTCCGTT	5F-adapter (5FA) – *kpigr1*
***Kpigr1*-3F**	ggtattccaattccattcgggaacaccacc-GACTGGTCGTCGAGCTAGTCCTG	3R-adapter (3RA) – *kpigr1*
***Kpigr1*-3R**	tgtgagcggataacaatttcacacaggaaaca-gggcccgctagcatttaaatgtttaaac-GGTGTCCAGCTCAAAGTGGCGC	pRS426 – MCS – *kpigr1*
***Kpigr2*-5F**	gttgggtaacgccagggttttcccagtcacga-ccgcgggtttaaacggtaccatttaaat-ATGGTCTGCGCCAACGACAAAGAC	pRS426 – MCS – *kpigr2*
***Kpigr2*-5R**	gtcttaagatcgcgaacaagacgtcgctcc-GTTATGGACTAGCAGCTATAGTGAGAGTCG	5F-adapter (5FA) – *kpigr2*
***Kpigr2*-3F**	ggtattccaattccattcgggaacaccacc-TCACGGTGATGACTGTCTGACGC	3R-adapter (3RA) – *kpigr2*
***Kpigr2*-3R**	tgtgagcggataacaatttcacacaggaaaca-ggtaccccgcggatttaaatgtttaaac-CACTGAGGGTGGTGTTGTCTGCC	pRS426 – MCS – *kpigr2*
**T*gluc*-3RAM-R**	ggtggtgttcccgaatggaattggaatacc-ttaattaa-ATCTTGTTGGGGGGAAGGGGTTG	3R-adapter – *Pac*I –T*gluc*
**P*trpC*-hiR**	GTGCTCACCGCCTGGACGACTAAACC	P*trpC* in R cassettes
***OgfpC*-P*tet*-F**	ccgcttgagcagacatcaccgtttcc-ATGGACAAGCAAAAAAATGGAATC	P*tet* – *gfpC*
***Glinker*-P*tet*-F**	ccgcttgagcagacatcaccgtttcc-ATGGAGCTCAGCGGCCGCCGC	P*tet* – linker-*gfpC/N*
***Ogfp*-P*tet*-F**	ccgcttgagcagacatcaccgtttcc-ATGGTTTCCAAGGGTGAGGTAAG	P*tet* – *gfp(N)*
***OgfpC*-R1**	ctaatcatacatcttatctacatacg-CTA-TTTGTAAAGTTCATCCATTCCC	T*gluc* – STOP – *ogfpC* $^{\Delta NotI}$
***OgfpC*-R2**	cctaatcatacatcttatctacatacg-ctat-gcggccgc-accagggcctccaagagatgcgtagtttggaacattataagggtatgggcctccacc-TTTGTAAAGTTCATCCATTCCCAA	T*gluc* – STOP – *Not*I – 19-aa linker – *ogfpC*
**T*trpC*-3RAM-R**	ggtggtgttcccgaatggaattggaatacc-ttaattaa-TCGAGTGGAGATGTGGAGTGGG	3R-adapter – *Pac*I –T*trpC*
**P*gpdA*-*Spe*I-P*trpC*-F**	gcccaaaaaatgctccttcaatatc-ACTAGT-ACAGTGACCGGTGACTCTT	P*trpC* – *Spe*I – P*gpdA*
***Kpwcl1*-P*tet*-F**	ccgcttgagcagacatcaccgtttcc-ATGAACGGCATGTCGAATATGTAC	P*tet* – *kpwcl1*
**P*gal1*X*P2A*XT*cyc1***	aggagaaaaaaccccggattctaga-ggcgcgccatg-ggctctggcgccaccaacttctctctcctcaaacaggct ggcgacgttgaagaaaaccctggccc-ttaatta-catgtaattagttatgtcacgctta	P*gal1* – *Asc*I/ATG – *P2A* – STOP/*Pac*I – T*cyc1* (ds)
***Kpgps1*-P*gal1*-F**	aggagaaaaaaccccggattctaga-ATGTCTCCTTCGACCACGTTGAC	P*gal1* – *kpgps1*

Name	Sequence (5'→ 3')	Features (5'→ 3')
***Kpgps1*-P*tet*-F**	cccgcttgagcagacatcaccgtttcc-ATGTCTCCTTCGACCACGTTGAC	P*tet* – *kpgps1*
***Kpgps1*-P2A-R**	gagagaagttggtggcgccagagcc-CTGCTTGTTATGCTGGCTTATTAGTTCC	P2A – *kpgps1*
***Kpphs1*-P*gal1*-F**	aggagaaaaaaccccggattctaga-ATGTACGACTATGCCTGGGTCCATG	P*gal1* – *kpphs1*
***Kpphs1*-P*tet*-F**	cccgcttgagcagacatcaccgtttcc-ATGTACGACTATGCCTGGGTCC	P*tet* – *kpphs1*
***Kpphs1*-P2A-F**	cgacgttgaagaaaaccctggccct-ATGTACGACTATGCCTGGGT	P2A – *kpphs1*
***Kpphs1*-Δstop-R**	CACGCTGCCTCTGCGCCCAGC	*kpphs1*
***Kpphd1*-ATG-F**	ATGGAGAAGCCTAAGAGTGTCATAG	*kpphd1*
***Kpphd1*-T*gluc*-R**	tcatacatcttatctacatacg-CTATCTAGTTTGTCTTGAGAAAAACAGC	T*gluc* – *kpphd1*
***Kpphd1*-T*cyc1*-R**	taagcgtgacataactaattacatg-CTATCTAGTTTGTCTTGAGAAAAACAGC	T*cyc1* – *kpphd1*
***Kpphs1*-2A-*phd1*-R**	ctatgacactcttaggcttctccat-agggccagggttttcttcaacgtcgccagcctgtttgaggagagagaagttggtggcgccagagcc-cacgctgcctctgcgcccagcc	*kpphd1* – P2A – *kpphs1* (as ss oligo in assembly)
***Kppks1*-P*tet*-F1**	ccgcttgagcagacatcaccgtttcc-ATGGAGGAAGTCTACGTGTTCGG	P*tet* – *kppks1*
***Kppks1*-T*gluc*-R**	taatcatacatcttatctacatacg-TTAGCCCTGAATGGCTTCACGAATG	T*gluc* – *kppks1*
***Kpura3*-P*tet*-F**	ccgcttgagcagACATCACCGTTTcc-ATGGCTTCGAGGAGTGCCGTACCG	P*tet* – *kpura3*
***Kpura3*-T*gluc*-R1**	cctaatcatacatcttatctacatacg-TCAGCCGACTCTCGCCTTGTACG	T*gluc* – *kpura3*
**pRS42X-sF1**	GCTGGCTTAACTATGCGGCATC	upstream of yeast gene
**pRS42X-sR1**	GCGGTATTTTCTCCTTACGCATCTG	downstream of yeast gene
**P*gpdA*-ATG-R**	CATAAGCTTAGATCTGTAGCTGTTAGTC	P*gpdA*
***Dsred*-P*gpdA*-F**	gactaacagctacagatctaagcttcc-ATGGCCTCCTCCGAGGACG	P*gpdA* – *dsred*
**T*trpC*-*niaD5*-R**	ccgtagctattagtaactggag-ggatcc-TCGAGTGGAGATGTGGAGTGGG	*bcniaD*-5' – *Bam*HI – T*trpC*
***Smh2b*-P*gpdA*-F**	gactaacagctacagatctaagcttcc-ATGGCTCCCAAGCCCGCCGAC	P*gpdA* – *smh2b*
***Smh2b*-*mch*-R**	tacttacctcgcccttgcttaccat-ATCGATAGAGTACTTGGTAACGGC	*mch* – *smh2b*
***Mch*-F1**	ccATGGTAAGCAAGGGCGAGGT	*mch*
***Mch*-*Not*I-R1**	gttgacatggagctattaaatca-ctaa-gcggccgc-TTTGTAGAGTTCATCCATTC	T*trpC* – STOP – *Not*I – *mch*
***Smh2b*-P*oliC*-F**	ccatcacatcacaatcgatccaacc-ATGGCTCCCAAGCCCGCCGAC	P*oliC* – *smh2b*
***Smh2b*-*gfp*-R**	tacttacctcacccttggaaaccat-ATCGATAGAGTACTTGGTAACGGC	*gfp* – *smh2b*
**P*oliC*-*Spe*I-F1**	gcccaaaaagtgctccttcaatgtc-actagt-TGCAGCTGTGGAGCCGCATTCCC	P*trpC* – *Spe*I – P*oliC*
**P*trpC*-*Spe*I-F1**	actagtGATATTGAAGGAGCATTTTTTGGGC	*Spe*I – P*trpC*
***Ogfp*-T*trpC*-R**	gttgacatggagctattaaatca-ctaa-gcggccgc-TTTGTAAAGTTCATCCATTCCC	T*trpC* – STOP – *Not*I – *ogfp*
**T*trpC*-F1**	gcggccgctTAGTGATTTAATAGCTCCATGTCAAC	*Not*I – STOP – T*trpC*-F
***Kpigr1*-RF-F1**	GCGGTTACCGAAGGATCTTTGCAAG	*kpigr1*-5' flank (LH)

Name	Sequence (5'➔ 3')	Features (5'➔ 3')
***Kpigr1*-RF-R1**	GTTCTGTAATCTTCGCTCGTACAACGG	*kpigr1*-3' flank (LH)
***Kpigr2*-RF-F1**	GCCTGCCAGAGTTCGGATTACCAG	*kpigr2*-5' flank (LH)
***Kpigr2*-RF-R1**	GACCTGAGCTACAGGCCTCGATCG	*kpigr2*-3' flank (LH)
**T*niaD-kpigr1*-SH5F**	ctgccgctcggaacgttccatccggtcctgtctgatccatccactcccaacggaccgtactctccgcgta-GCATTGGATTAATAATTGTTGCTAAGCGAG	*kpigr1*-5' (70 nt) – T*niaD*
**T*gluc-kpigr1*-SH3R**	gtagggcatggcggtgagtgaggctcaacggtgatcgcgatcgatctggcaaaacaggactagctcgacgaccagtc-ATCTTGTTGGGGGGAAGGGGTTG	*kpigr1*-3' (77 nt) – T*gluc*
**T*niiA-kpigr2*-SH5F**	cgaacactaggcgatgccgacaacacaacagagtcaccgtcgactctcactatagctgctagtccataac-AGATGCTGCTGGCAAGGTTAC	*kpigr2*-5' (70 nt) – T*niiA*
**T*gluc-kpigr2*-SH3R**	ttgattgtgcacctacggaaagaactagaaatacatcctgttggccgagctggtgcgtcagacagtcatcaccgtga-ATCTTGTTGGGGGGAAGGGGTTG	*kpigr2*-3' (77 nt) – T*gluc*
**T*niaD-kpigr3*-SH5F**	aacccagcacctctattcgtagtccgtctagcgacttacactcaccgtcttgcgttgatgtgaacctgtt-GCATTGGATTAATAATTGTTGCTAAGCGAG	*kpigr3*-5' (70 nt) – T*niaD*
**T*gluc-kpigr3*-SH3R**	tacagtgccactagcttctgacaagagctataatgtaccgacaggtgttcgatgttgacttaagtcacgtgcactct-ATCTTGTTGGGGGGAAGGGGTTG	*kpigr3*-3' (77 nt) – T*gluc*
**T*niaD-kpigr4*-SH5F**	tgggttcaggcatgtaattatcggttggctgcaggctgccaggtatgcctgggtccgcgcaaacctggga-GCATTGGATTAATAATTGTTGCTAAGCGAG	*kpigr4*-5' (70 nt) – T*niaD*
**T*gluc-kpigr4*-SH3R**	cttacattcgaggcgatgaaactagcatccataattaattacatcggctaaaaaccgttgaagtgcagatgccgacg-ATCTTGTTGGGGGGAAGGGGTTG	*kpigr4*-3' (77nt) – T*gluc*
**T*niaD-kpigr5*-SH5F**	ttgtgaggagcagttaaatcgccgatgataggtttttttgacagtcaagcaagcattaccgttcgtcggg-GCATTGGATTAATAATTGTTGCTAAGCGAG	*kpigr5*-5' (70 nt) – T*niaD*
**T*gluc-kpigr5*-SH3R**	ttggctccacatcgcttagagatcttgccatcttcgcgggatctgcagcgcaatcgcgcagagaggagacacctgcg-ATCTTGTTGGGGGGAAGGGGTTG	*kpigr5*-3' (77 nt) – T*gluc*
***Kppks1*-RT5F**	gatcagcccttcttttgtttttctgctcgttaagaaccgcacccgaagtacgtcgacactcattcacatttcact-GCTAAGCGAGCGGGAGCTATCG	*kppks1*-5' (76 nt) – T*niaD*
***Kppks1*-RT3R**	gttgaaacaggttgcagtagttgatccagacaacaccatgatatgccagtcaagtgagtaggttcgtgtggttgc-GAATCGGGAATGCGGCTCCACAG	*kppks1*-3' (75 nt) – *PoliC*
***Kpphs1*-RT5F**	tcacgttactcaccaccaaagccgtgattcttgatttcgcgtagacgtctgcacatcaacttgcgcatcgcgatc-GCTAAGCGAGCGGGAGCTATCG	*kpphs1*-5' (76 nt) – T*niaD*
***Kpphs1*-RT3R**	cgcggcgactgcattactattcttacgttgaaggcccgctacacaagcagaagtcgagagccgttagacgcagat-GAATCGGGAATGCGGCTCCACAG	*kpphs1*-3' (75 nt) – *PoliC*
***Kpphd1*-RT3R**	gctgcttttgggaataccaatcgctggttcttattggatgtcggagaatgaggtccagccgtctattgatcaacg-GAATCGGGAATGCGGCTCCACAG	*kpphd1*-3' (75 nt) – *PoliC*
***Kpade2*-RT5F**	aactgcccaactggcccaaagttccgccctactttgggctcacccacccaccttgtaactacctgtagtaacttt-GCTAAGCGAGCGGGAGCTATCG	*kpade2*-5' (75 nt) – T*niaD*
***Kpade2*-RT3R**	cgtggtggccgcatacgtacgcatcgaaccgagttccttcccgctccgctatcactatcctttcagccctactgc-GAATCGGGAATGCGGCTCCACAG	*kpade2*-3' (75 nt) – *PoliC*
**T*niaD-kppks1*-SH5F**	agcgtccactctttccgcagcaagtatcccagtagaccagcagatgcttctcagcttgatcactcctgc-GCATTGGATTAATAATTGTTGCTAAGCGAG	*kppks1*-5' (69 nt) – T*niaD*
**T*gluc-kppks1*-SH3R**	tgttgaaacaggttgcagtagttgatccagacaacaccatgatatgccagtcaagtgagtaggttcgtgtggttgc-ATCTTGTTGGGGGGAAGGGGTTG	*kppks1*-3' (76 nt) – T*gluc*
**T*niaD-kpphs1*-SH5F**	tactcaccaccaaagccgtgattcttgatttcgcgtagacgtctgcacatcaacttgcgcatcgcgatcc-GCATTGGATTAATAATTGTTGCTAAGCGAG	*kpphs1*-5' (70 nt) – T*niaD*
**T*trpC-kpphs1*-SH3R**	gcgcggcgactgcattactattcttacgttgaaggcccgctacacaagcagaagtcgagagccgttagacgcagat-TCGAGTGGAGATGTGGAGTGGG	*kpphs1*-3' (76 nt) – T*trpC*
**T*trpC-kpade2*-SH3R**	cgtggtggccgcatacgtacgcatcgaaccgagttccttcccgctccgctatcactatcctttcagccctactgc-TCGAGTGGAGATGTGGAGTGGG	*kpade2*-3' (75 nt) – T*trpC*

Lowercase letters – 5' sequences (overhangs) for cloning (*in-vivo* or *in-vitro* DNA assembly) or for mediating homologous recombination and additional sequences
Uppercase letters – 3' sequences annealing to the template DNA

Table S5 Oligonucleotides used for sequencing and diagnostic PCR in this study.

Name	Sequence (5'→ 3')	Binding site
**P*afU3*-sF1**	GCTTGAGGTTAGCGCACTCGCTAG	P*afU3* from *A. fumigatus* in pAMA/tRNA-*goi* PSX(-*goi* PSX)
**P*tef1*-sR1**	CGTTCGAGAGCATGATCAGCAC	P*tef1* from *A. nidulans* in pAMA/tRNA-*goi* PSX(-*goi* PSX)
**pRS426-s3R2**	CATTAATGCAGCTGGCACGACAGG	upstream of cloning site/MCS in pRS42X derivatives
**pRS426-s5F2**	GTAGCGGTCACGCTGCGCGTAACC	downstream of cloning site/MCS in pRS42X derivatives
**P*gal1*-sF1**	GTCGCGTTCCTGAAACGCAGATG	P*gal1* from *S. cerevisiae* in p42X GAL derivatives
**T*cyc1*-sR1**	GGACCTAGACTTCAGGTTGTCTAAC	T*cyc1* from *S. cerevisiae* in p42X GAL derivatives
**P*oliC*-sF2**	GGGAGACGTATTTAGGTGCTAGGG	P*oliC* from *A. nidulans* in pNXR-OXX, pIGRXR-OXX
**P*oliC*-sR1**	GTACTGCCCCGCTTAGTGGCAG	P*oliC* from *A. nidulans* in pNXR-OXX, pIGRXR-OXX
**P*gpdA*-hiR**	GCGCAGTGGCGCTTATTACTCAG	P*gpdA* from *A. nidulans* in pNXR-GXX, pIGRXR-GXX
**P*gpdA*-sF1**	GTTGACAAGGTCGTTGCGTCAG	P*gpdA* from *A. nidulans* in pNXR-GXX, pIGRXR-GXX
**P*tet*-sF1**	GTCGAGCTCCCCATCTTCAGTATATTC	P*tet* (synthetic *tetO7+Pmin*) in pIGRXR-TXX
**T*gluc*-hiF**	CATACGTACATCTGATTTGACAACC	T*gluc* from *B. cinerea* in pNDR-XXX, pIGRXR-XXX
**T*gluc*-sR2**	CCGCCCTCTTTTGTCTTCCGC	T*gluc* from *B. cinerea* in pNDR-XXX, pIGRXR-XXX
**T*trpC*-hiF**	ACCCAGAATGCACAGGTACACTTG	T*trpC* from *A. nidulans* in pNXR-XXT, pIGRXR-XXT
**T*trpC*-sR2**	GGAGCCTGAAGGGCGTACTAGGG	T*trpC* from *A. nidulans* in pNXR-XXT, pIGRXR-XXT
**5FA-hiR**	GTCTTAAGATCGCGAACAAGACGTCG	synthetic 5F-adapter (5FA) in pIGRXR(-XXX)
**3RA-hiF**	GTATTCCAATTCCATTCGGGAACACCAC	synthetic 3R-adapter (3RA) in pIGRXR(-XXX)
***P2A*-sF1**	GCTCTGGCGCCACCAACTTCTC	*P2A* (synthetic) for polycistronic transcripts
***P2A*-sR1**	GGCCAGGGTTTTCTTCAACGTCG	*P2A* (synthetic) for polycistronic transcripts
***Ogfp*-sR1**	CGTCTCCCTCACCCTCTCCG	*gfp* optimized for *B. cinerea* in pNXR-XGX, pIGRXR-XGX
***Ogfp*-sF1**	GGTGATGGTCCAGTCTTGCTC	*gfp* optimized for *B. cinerea* in pNXR-XGX, pIGRXR-XGX
***OgfpN*-sF1**	CAACCGTATTGAGTTGAAGGGC	*gfp* optimized for *B. cinerea* in pNXR-XGX, pIGRXR-XGX
***OgfpC*-sR1**	CTGCCAATTGAACAGAGCCATCC	*gfp* optimized for *B. cinerea* in pNXR-XGX, pIGRXR-XGX
***Omch*-sR2**	GTCGGCCCTCACCTTCGCCCTCG	*mch* optimized for *B. cinerea* in pNXR-XCX, pIGRXR-XCX
***Dsred*-sR3**	GCCCTTGGTCACCTTCAGCTTG	*dsred* in pNXR-XDX, pIGRXR-XDX
***Smh2b*-sR1**	GGCAACTCACCCTTGTAGATGTAG	*h2b* from *S. macrospora* in pNDR-XNXX
***Hph*-hiF**	GTCTGGACCGATGGCTGTGTAGAAG	*hph* [hygromycin resistance (hygR, H) cassette]
***Hph*-hiR**	GACAGACGTCGCGGTGAGTTCAG	*hph* [hygromycin resistance (hygR, H) cassette]
***Nat1*-hiF**	CGGCGAGCAGGCGCTCTACATGAGC	*nat1* [nourseothricin resistance (natR, N) cassette]
***Nat1*-hiR**	GTACCGGTAAGCCGTGTCGTCGAG	*nat1* [nourseothricin resistance (natR, N) cassette]
***NptII*-hiR**	GCCGAATAGCCTCTCCACCCAAG	*nptII* [geneticin/G418 resistance (genR, G) cassette]
***NptII*-hiF**	GCCTTCTATCGCCTTCTTGACGAG	*nptII* [geneticin/G418 resistance (genR, G) cassette]
***Bar*-hiR**	GAAGTTGACCGTGCTTGTCTCGATG	*bar* [glufosinate resistance (baR, P) cassette]
***Bar*-hiF**	GAACTGGCATGACGTGGGTTTCTG	*bar* [glufosinate resistance (baR, P) cassette]
***Sur*-hiR**	GCTTGAGAGTCGAGATGGTTCGTG	*sur* [chlorimuron ethyl resistance (suR, S) cassette]
***Sur*-hiF**	GCACGAGTTCATCACGTTTGATGC	*sur* [chlorimuron ethyl resistance (suR, S) cassette]
***Kpigr1*-hi5F**	GATGGGCCAGGCGTTGTTCATTC	upstream of *kpigr1*-LH5' flank in *K. petricola* genome
***Kpigr1*-hi3R**	GGCAGCTGCTAGACGAATTAGTGC	downstream of *kpigr1*-LH3' flank in *K. petricola* genome
***Kpigr1*-wtF1**	GCCTCTCAAATTCCTCCGCGCAG	upstream of *kpigr1*-SH5' flank in *K. petricola* genome
***Kpigr1*-wtR1**	GACTCGTGAAGAAGCAGCCCACAC	downstream of *kpigr1*-SH3' flank in *K. petricola* genome
***Kpigr2*-hi5F**	GAGCGATGTCGTCTCGGAAGATTAC	upstream of *kpigr2*-LH5' flank in *K. petricola* genome
***Kpigr2*-hi3R**	GAGCAGCTAAGGTTGAGGAGCCC	downstream of *kpigr2*-LH3' flank in *K. petricola* genome
***Kpigr2*-wtF1**	GGCAACTCAGACGCAATGGAGCC	upstream of *kpigr2*-SH5' flank in *K. petricola* genome
***Kpigr2*-wtR1**	CCTGAGCTACAGGCCTCGATCG	downstream of *kpigr2*-SH3' flank in *K. petricola* genome
***Kpigr3*-hi5F**	GCTCCTTCTCACGTACCACCAGACG	upstream of *kpigr3*-SH5' flank in *K. petricola* genome
***Kpigr3*-hi3R**	GCCGAGTCCAATTGGTTGATCGATC	downstream of *kpigr3*-SH3' flank in *K. petricola* genome

Name	Sequence (5'➔ 3')	Binding site
***Kpigr4*-hi5F**	GGTCAAGCACCGATTCTTCCGCG	upstream of *kpigr4*-SH5' flank in *K. petricola* genome
***Kpigr4*-hi3R**	GTAAGGAGAGTCCCGGTCGACGTTG	downstream of *kpigr4*-SH3' flank in *K. petricola* genome
***Kpigr5*-hi5F**	GCATGCGAAGTACGCTTAGCGAG	upstream of *kpigr5*-SH5' flank in *K. petricola* genome
***Kpigr5*-hi3R**	GCTCCTCGGAACGGAGTCGAACATG	downstream of *kpigr5*-SH3' flank in *K. petricola* genome
***Kppks1*-hi5F3**	GTGGATCGAAACATTGGTTCTGCTG	upstream of *kppks1*-SH5' flank in *K. petricola* genome
***Kppks1*-hi3R3**	GCGCCGTCTATAACCTTTGCAATAG	downstream of *kppks1*-SH3' flank in *K. petricola* genome
***Kppks1*-wtF2**	GCCGATCTGGCATACACCACCAC	in coding region of *kppks1*
***Kppks1*-wtR2**	GTCCGAGACGCCGTTGATGCATG	in coding region of *kppks1*
***Kpphs1*-SH-hi5F**	GGTGCTAAGCTTTGCCAGCCTCG	upstream of *kpphs1*-SH5' flank in *K. petricola* genome
***Kpphs1*-SH-hi3R**	GTGGGTTACAGGCTACAATGTCAGG	downstream of *kpphs1*-SH3' flank in *K. petricola* genome
***Kpphd1*-SH-hi3R**	GGTTGCTGTTCAACGAGGCCAATG	downstream of *kpphd1*-SH3' flank in *K. petricola* genome
***Kpphs1*-wtF1**	GTTCGATGTACCTGCCGAGGAGTTG	in coding region of *kpphs1*
***Kpphs1*-wtR1**	CTCGGCTGCACTAGCAGCATCATC	in coding region of *kpphs1*
***Kpade2*-hi5F**	CCATTGGCAGGTACAAGCCACTG	upstream of *kpade2*-SH5' flank in *K. petricola* genome
***Kpade2*-hi3R**	GGAATACGCGGGTATGCAGTCGC	downstream of *kpade2*-SH3' flank in *K. petricola* genome
***Kpade2*-wtF2**	GCACAGAATACAACCTTCGTGG	in coding region of *kpade2*
***Kpade2*-wtR2**	GATCGTGTAATGGCCTGAGT	in coding region of *kpade2*

Table S6 Plasmids cloned in this study.

**Name** [ID] (size)	**Entry plasmid**	**Amplicon (s)**				**Method**
**pNDG-OGG v2**<br>**[pEC0270]** (10.851 kb)	**pNDN-OGG**<br>(Schumacher 2012)<br>digested w/ *Kpn*I + *Sph*I	***nptII*-A** (0.707 kb)<br>primers: P*trpC*-sF2/*nptII*-mut-R1<br>template: pNDG-OGG v1 [pEC0119] (Erdmann et al. 2022)	***nptII*-B** (0.432 kb)<br>primers: *nptII*-mut-F1/T*niaD*-sR1<br>template: pNDG-OGG v1 [pEC0119] (Erdmann et al. 2022)			*In vivo*
**pNDS-OGG v2**<br>**[pEC0307]** (12.543 kb)	**pNDN-OGG**<br>(Schumacher 2012)<br>digested w/ *Kpn*I + *Sph*I	***sur*-1** (0.981 kb)<br>primers: P*trpC*-sF2/<br>*sur*-mut-R1; template:<br>pNDS-OGG v1 [pEC0128]	***sur*-2** (1.102 kb)<br>primers: *sur*-mut-F1/<br>*sur*-mut-R2; template:<br>pNDS-OGG v1 [pEC0128]	***sur*-3** (0.422 kb)<br>primers: *sur*-mut-F2/<br>*sur*-mut-R3; template:<br>pNDS-OGG v1 [pEC0128]	***sur*-4** (0.409 kb)<br>primers: *sur*-mut-F3/<br>T*niaD*-sR1; template:<br>pNDS-OGG v1 [pEC0128]	*In vivo*
**pIGR1G v2**<br>**[pEC0290]** (9.201 kb)	**pRS426$^{\Delta NcoI}$**<br>(Schumacher 2012)<br>digested w/ *Eco*RI + *Xho*I	**P*trpC*::*nptII*::T*niaD*** (1.610 kb)<br>primers: P*trpC*-3RAM-R2/T*niaD*-5FAM-R<br>template: pNDG-OGG v2 [pEC0270] (this study)	***kpigr1* 5' flank** (1.185 kb)<br>primers: *kpigr1*-5F/*kpigr1*-5R<br>template: *K. petricola* DNA	***kpigr1* 3' flank** (0.848 kb)<br>primers: *kpigr1*-3F/*kpigr1*-3R<br>template: *K. petricola* DNA		*In vivo*
**pIGR2G v2**<br>**[pEC0291]** (9.676 kb)	**pRS426$^{\Delta NcoI}$**<br>(Schumacher 2012)<br>digested w/ *Eco*RI + *Xho*I	**P*trpC*::*nptII*::T*niaD*** (1.610 kb)<br>primers: P*trpC*-3RAM-R2/T*niaD*-5FAM-R<br>template: pNDG-OGG v2 [pEC0270] (this study)	***kpigr2* 5' flank** (0.917 kb)<br>primers: *kpigr2*-5F/*kpigr2*-5R<br>template: *K. petricola* DNA	***kpigr2* 3' flank** (1.592 kb)<br>primers: *kpigr2*-3F/*kpigr2*-3R<br>template: *K. petricola* DNA		*In vivo*
**pIGR1H-TGG**<br>**[pEC0362]** (13.139 kb)	**pIGR1H** [pEC0286]<br>(Erdmann et al. 2022)<br>digested w/ *Pac*I	**P*oliC*::TET::*gfp*::T*gluc*** (3.787 kb)<br>primers: T*gluc*-3RAM-R/P*trpC*-hiR<br>template: pNAH-OTGG (Janevska et al. 2017)	n/a			*In vivo*
**pIGR2H-TGG**<br>**[pEC0363]** (13.614 kb)	**pIGR2H** [pEC0287]<br>(Erdmann et al. 2022)<br>digested w/ *Pac*I	**P*oliC*::TET::*gfp*::T*gluc*** (3.787 kb)<br>primers: T*gluc*-3RAM-R/P*trpC*-hiR<br>template: pNAH-OTGG (Janevska et al. 2017)	n/a			*In vivo*
**pIGR1N-TGG**<br>**[pEC0364]** (12.671 kb)	**pIGR1N** [pEC0288]<br>(Erdmann et al. 2022)<br>digested w/ *Pac*I	**P*oliC*::TET::*gfp*::T*gluc*** (3.787 kb)<br>primers: T*gluc*-3RAM-R/P*trpC*-hiR<br>template: pNAH-OTGG (Janevska et al. 2017)	n/a			*In vivo*
**pIGR2N-TGG**<br>**[pEC0365]** (13.146 kb)	**pIGR2N** [pEC0289]<br>(Erdmann et al. 2022)<br>digested w/ *Pac*I	**P*oliC*::TET::*gfp*::T*gluc*** (3.787 kb)<br>primers: T*gluc*-3RAM-R/P*trpC*-hiR<br>template: pNAH-OTGG (Janevska et al. 2017)	n/a			*In vivo*
**pIGR1G-TGG**<br>**[pEC0366]** (12.896 kb)	**pIGR1G v2** [pEC0290]<br>(this study)<br>digested w/ *Pac*I	**P*oliC*::TET::*gfp*::T*gluc*** (3.787 kb)<br>primers: T*gluc*-3RAM-R/P*trpC*-hiR<br>template: pNAH-OTGG (Janevska et al. 2017)	n/a			*In vivo*
**pIGR2G-TGG**<br>**[pEC0367]** (13.371 kb)	**pIGR2G v2** [pEC0291]<br>(this study)<br>digested w/ *Pac*I	**P*oliC*::TET::*gfp*::T*gluc*** (3.787 kb)<br>primers: T*gluc*-3RAM-R/P*trpC*-hiR<br>template: pNAH-OTGG (Janevska et al. 2017)	n/a			*In vivo*
**pIGR1P-TGG**<br>**[pEC0368]** (12.653 kb)	**pIGR1P** [pEC0292]<br>(Erdmann et al. 2022)<br>digested w/ *Pac*I	**P*oliC*::TET::*gfp*::T*gluc*** (3.787 kb)<br>primers: T*gluc*-3RAM-R/P*trpC*-hiR<br>template: pNAH-OTGG (Janevska et al. 2017)	n/a			*In vivo*

**Name** [ID] (size)	**Entry plasmid**	**Amplicon (s)**		**Method**
**pIGR2P-TGG [pEC0369]** (13.128 kb)	**pIGR2P** [pEC0293]<br>(Erdmann et al. 2022)<br>digested w/ *Pac*I	**P*oliC*::TET::*gfp*::T*gluc*** (3.787 kb)<br>primers: T*gluc*-3RAM-R/P*trpC*-hiR<br>template: pNAH-OTGG (Janevska et al. 2017)	n/a	*In vivo*
**pIGR1S-TGG [pEC0231]** (14.588 kb)	**pIGR1S** [pEC0179]<br>(Erdmann et al. 2022)<br>digested w/ *Pac*I	**P*oliC*::TET::*gfp*::T*gluc*** (3.787 kb)<br>primers: T*gluc*-3RAM-R/P*trpC*-hiR<br>template: pNAH-OTGG (Janevska et al. 2017)	n/a	*In vivo*
**pIGR2S-TGG [pEC0235]** (15.063 kb)	**pIGR2S** [pEC0180]<br>(Erdmann et al. 2022)<br>digested w/ *Pac*I	**P*oliC*::TET::*gfp*::T*gluc*** (3.787 kb)<br>primers: T*gluc*-3RAM-R/P*trpC*-hiR<br>template: pNAH-OTGG (Janevska et al. 2017)	n/a	*In vivo*
**pIGR1H-TGCGn [pEC0435]** (12.683 kb)	**pIGR1H-TGG** [pEC0362]<br>(this study)<br>digested w/ *Nco*I + *Not*I	***gfpC-n*** (0.376 kb)<br>primers: *ogfpC*-P*tet*-F/*ogfpC*-R2<br>template: pNAH-OGCGn (Schumacher 2012)	n/a	*In vitro*
**pIGR1H-TGCGc [pEC0436]** (12.683 kb)	**pIGR1H-TGG** [pEC0362]<br>(this study)<br>digested w/ *Nco*I + *Not*I	***gfpC-c*** (0.376 kb)<br>primers: *Glinker*-P*tet*-F/*ogfpC*-R1<br>template: pNAH-OGCGc (Schumacher 2012)	n/a	*In vitro*
**pIGR2N-TGNTn [pEC0437]** (13.061 kb)	**pIGR2N-TGG** [pEC0365]<br>(this study)<br>digested w/ *Nco*I + *Pac*I	***gfpN-n*** (1.233 kb)<br>primers: *ogfp*-P*tet*-F/T*trpC*-3RAM-R<br>template: pNDN-AGNTn (Schumacher 2012)	n/a	*In vitro*
**pIGR2N-TGNTc [pEC0438]** (13.040 kb)	**pIGR2N-TGG** [pEC0365]<br>(this study)<br>digested w/ *Nco*I + *Pac*I	***gfpN-c*** (1.238 kb)<br>primers: *Glinker*-P*tet*-F/T*trpC*-3RAM-R<br>template: pNDN-AGNTc (Schumacher 2012)	n/a	*In vitro*
**pIGR1H-OE::W1GC [pEC0445]** (14.390 kb)	**pIGR1H** [pEC0286]<br>(Erdmann et al. 2022)<br>digested w/ *Pac*I	**P*gpdA*::*kpwcl1-gfpC*::T*gluc*** (5.009 kb)<br>primers: P*gpdA*-SpeI-P*trpC*-F/T*gluc*-3RAM-R<br>template: pNAH-G^{W1}GCG [pEC0045] (Erdmann et al. 2022)	n/a	*In vivo*
**pIGR2N-OE::GNW2 [pEC0446]** (13.067 kb)	**pIGR2N** [pEC0289]<br>(Erdmann et al. 2022)<br>digested w/ *Pac*I	**P*gpdA*::*gfpN-kpwcl2*::T*trpC*** (3.679 kb)<br>primers: P*gpdA*-SpeI-P*trpC*-F/T*trpC*-3RAM-R<br>template: pNDN-GGN^{W2}T [pEC0130] (Erdmann et al. 2022)	n/a	*In vivo*
**pIGR1H-TET::W1GC [pEC0469]** (15.928 kb)	**pIGR1H-TGG** [pEC0362]<br>(this study)<br>digested w/ *Nco*I + *Not*I	***kpwcl1-gfpC*** (3.621 kb)<br>primers: *kpwcl1*-P*tet*-F/*ogfpC*-R1<br>template: pNAH-G^{W1}GCG [pEC0045] (Erdmann et al. 2022)	n/a	*In vitro*
**pIGR2N-TET::GNW2 [pEC0440]** (14.603 kb)	**pIGR2N-TGG** [pEC0365]<br>(this study)<br>digested w/ *Not*I	***gfpN-kpwcl2*** (2.801 kb)<br>primers: *ogfp*-P*tet*-F/T*trpC*-3RAM-R<br>template: pNDN-GGN^{W2}T [pEC0130] (Erdmann et al. 2022)	n/a	*In vivo*
**p426-GAL1::P2A [pEC0248]** (6.432 kb)	**p426 GAL1**<br>(Mumberg et al. 1994)<br>digested w/ *Spe*I + *Xho*I	***Asc*I-start-*2A*-stop-*Pac*I** (0.134 kb)<br>= synthetic double-stranded DNA fragment [P*gal1*X*P2A*XT*cyc1*] (this study)		*In vivo*

Name [ID] (size)	Entry plasmid	Amplicon (s)				Method
**pURA-GAL::PP [pEC0348]** (9.888 kb)	**p426 GAL1**<br>(Mumberg et al. 1994)<br>digested w/ *Spe*I + *Xho*I	***kpphs1*** (1.816 kb)<br>primers: *kpphs1*-P*gal1*-F/*kpphs1*-Δstop-R; template: *K. petricola* cDNA	***kpphd1*** (1.708 kb)<br>primers: *kpphd1*-ATG-F/*kpphd1*-T*cyc1*-R; template: *K. petricola* cDNA	***kpphs1-2A-kpphd1*-R** (0.115 kb)<br>= synthetic single-stranded DNA oligonucleotide		*In vivo*
**pURA-GAL::G^{P2A} [pEC0353]** (7.669 kb)	**p426-GAL1::P2A**<br>[pEC0248] (this study)<br>digested w/ *Asc*I	***kpgps1*** (1.248 kb)<br>primers: *kpgps1*-P*gal1*-F/*kpgps1*-*P2A*-R<br>template: *K. petricola* gDNA	n/a			*In vivo*
**pURA-GAL::GPP [pEC0354]** (11.202 kb)	**pURA-GAL::G^{P2A}**<br>[pEC0353] (this study)<br>digested w/ *Pac*I	***kpphs1-kpphd1*** (3.590 kb)<br>primers: *kpphs1*-*P2A*-F/*kpphd1*-T*cyc1*-R<br>template: pURA-GAL::PP [pEC0348] (this study)	n/a			*In vitro*
**pIGR2H-TET::PP [pEC0355]** (16.374 kb)	**pIGR2H-TGG** [pEC0363]<br>(this study)<br>digested w/ *Nco*I + *Not*I	***kpphs1-kpphd1*** (3.590 kb)<br>primers: *kpphs1*-P*tet*-F/*kpphd1*-T*gluc*-R<br>template: pURA-GAL::PP [pEC0348] (this study)	n/a			*In vivo*
**pIGR2H-TET::GPP [pEC0392]** (17.688 kb)	**pIGR2H-TGG** [pEC0363]<br>(this study)<br>digested w/ *Nco*I + *Not*I	***kpgps1-2A-phs1-2A-phd1*** (4.855 kb)<br>primers: *kpgps1*-P*tet*-F/*kpphd1*-T*gluc*-R<br>template: pURA-GAL::GPP [pEC0354] (this study)	n/a			*In vitro*
**pIGR2H-TET::*pks1* [pEC0394]** (19.383 kb)	**pIGR2H-TGG** [pEC0363]<br>(this study)<br>digested w/ *Nco*I + *Not*I	***kppks1*** $^{\Delta intron}$ (6.550 kb)<br>primers: *kppks1*-P*tet*-F1/*kppks1*-T*gluc*-R<br>template: pURA-*kppks1* [pEC0243] (Catanzaro et al., in preparation)	n/a			*In vitro*
**pIGR2H-TET::*ura3* [pEC0294]** (14.767 kb)	**pIGR2H-TGG** [pEC0363]<br>(this study)<br>digested w/ *Nco*I	***kpura3*** $^{\Delta intron}$ (0.863 kb)<br>primers: *kpura3*-P*tet*-F/*kpura3*-T*gluc*-R1<br>template: pLEU-*kpura3*-WT	***scleu2*** (2.332 kb)<br>primers: pRS42X-sF1/ pRS42X-sR1<br>template: pRS425 GAL1 (Mumberg et al. 1994)			*In vivo*
**pAMA/tRNA-*igr3*^{PS1} [pEC0419]** (16.449 kb)	**pFC332**<br>(Nødvig et al. 2015)<br>digested w/ *Pac*I	***kpigr3*^{PS1} tRNA-A** (0.589 kb)<br>primers: pFC334-F1/*kpigr3*-tRNA-PS1R<br>template: pFC902 (Nødvig et al. 2018)	***kpigr3*^{PS1} tRNA-B** (0.427 kb)<br>primers: *kpigr3*-tRNA-PS1F/pFC334-R1<br>template: pFC902 (Nødvig et al. 2018)			*In vitro*
**pAMA/tRNA-*igr4*^{PS1} [pEC0420]** (16.449 kb)	**pFC332**<br>(Nødvig et al. 2015)<br>digested w/ *Pac*I	***kpigr4*^{PS1} tRNA-A** (0.589 kb)<br>primers: pFC334-F1/*kpigr4*-tRNA-PS1R<br>template: pFC902 (Nødvig et al. 2018)	***kpigr4*^{PS1} tRNA-B** (0.427 kb)<br>primers: *kpigr4*-tRNA-PS1F/pFC334-R1<br>template: pFC902 (Nødvig et al. 2018)			*In vitro*
**pAMA/tRNA-*igr5*^{PS1} [pEC0421]** (16.449 kb)	**pFC332**<br>(Nødvig et al. 2015)<br>digested w/ *Pac*I	***kpigr5*^{PS1} tRNA-A** (0.589 kb)<br>primers: pFC334-F1/*kpigr5*-tRNA-PS1R<br>template: pFC902 (Nødvig et al. 2018)	***kpigr5*^{PS1} tRNA-B** (0.427 kb)<br>primers: *kpigr5*-tRNA-PS1F/pFC334-R1<br>template: pFC902 (Nødvig et al. 2018)			*In vitro*
**pAMA/tRNA-*pks1*^{PS2}-*phs1*^{PS1}-*ade2*^{PS3} [pEC0195]** (16.841 kb)	**pFC332**<br>(Nødvig et al. 2015)<br>digested w/ *Pac*I	***kppks1*^{PS2} tRNA** (0.589 kb)<br>primers: pFC334-F1/ *kppks1*-tRNA-PS2R template: pFC902	***kppks1*^{PS2}/*kpphs1*^{PS1} tRNA** (0.191 kb)<br>primers: *kppks1*-tRNA-PS2F/*kpphs1*-tRNA-PS1R<br>template: pFC902	***kpphs1*^{PS1}/*kpade2*^{PS3} tRNA** (0.191 kb)<br>primers: *kpphs1*-tRNA-PS1F/*kpade2*-tRNA-PS3R<br>template: pFC902	***kpade2*^{PS3} tRNA** (0.427 kb)<br>primers: *kpade2*-tRNA-PS3F/pFC334-R1<br>template: pFC902	*In vitro*

**Name** [ID] (size)	**Entry plasmid**	**Amplicon (s)**	**Method**
**pAMA/tRNA-*igr1*^{PS1}-*igr2*^{PS1}-*igr3*^{PS1} [pEC0467]** (16.841 kb)	**pFC332**<br>(Nødvig et al. 2015)<br>digested w/ *Pac*I	***kpigr1*^{PS1}-tRNA** (0.589 kb)<br>primers: pFC334-F1/*kpigr1*-tRNA-PS1R<br>template: pFC902<br><br>***kpigr1*^{PS1}/*kpigr2*^{PS1}**-tRNA (0.191 kb)<br>primers: *kpigr1*-tRNA-PS1F/*kpigr2*-tRNA-PS1R<br>template: pFC902<br><br>***kpigr2*^{PS1}/*kpigr3*^{PS1}-tRNA** (0.191 kb)<br>primers: *kpigr2*-tRNA-PS1F/*kpigr3*-tRNA-PS1R<br>template: pFC902<br><br>***kpigr3*^{PS1}-tRNA** (0.427 kb)<br>primers: *kpigr3*-tRNA-PS1F/pFC334-R1<br>template: pFC902	*In vitro*
**pAMA/tRNA-*igr1*^{PS1}-*igr2*^{PS1}-*igr3*^{PS1}-*igr4*^{PS1} [pEC0468]** (17.012 kb)	**pFC332**<br>(Nødvig et al. 2015)<br>digested w/ *Pac*I	***kpigr1*^{PS1}-tRNA** (0.589 kb)<br>primers: pFC334-F1/ *kpigr1*-tRNA-PS1R<br>template: pFC902<br><br>***kpigr1*^{PS1}/*kpigr2*^{PS1}-tRNA** (0.191 kb)<br>primers: *kpigr1*-tRNA-PS1F/ *kpigr2*-tRNA-PS1R<br>template: pFC902<br><br>***kpigr2*^{PS1}/*kpigr3*^{PS1}-tRNA** (0.191 kb)<br>primers: *kpigr2*-tRNA-PS1F/ *kpigr3*-tRNA-PS1R<br>template: pFC902<br><br>***kpigr3*^{PS1}/*kpigr4*^{PS1}-tRNA** (0.191 kb)<br>primers: *kpigr3*-tRNA-PS1F/ *kpigr4*-tRNA-PS1R<br>template: pFC902<br><br>***kpigr4*^{PS1}-tRNA** (0.427 kb)<br>primers: *kpigr4*-tRNA-PS1F/ pFC334-R1<br>template: pFC902	*In vitro*
**pAMA/tRNA-*pks1*^{PS2}-*pks1*^{PS5}-*phs1*^{PS1}-*phs1*^{PS2}-*ade2*^{PS2}-*ade2*^{PS5} [pEC0389]** (17.354 kb)	**pFC332**<br>(Nødvig et al. 2015)<br>digested w/ *Pac*I	***kppks1*^{PS2}-tRNA** (0.589 kb)<br>primers: pFC334-F1/ *kppks1*-tRNA-PS2R<br>template: pFC902<br><br>***kppks1*^{PS2}/*kppks1*^{PS5}-tRNA** (0.191 kb)<br>primers: *kppks1*-tRNA-PS2F/ *kppks1*-tRNA-PS5R; template: pFC902<br><br>***kppks1*^{PS2}/*kpphs1*^{PS1}-tRNA** (0.191 kb)<br>primers: *kppks1*-tRNA-PS5F/ *kpphs1*-tRNA-PS1R; template: pFC902<br><br>***kpphs1*^{PS1}/*kpphs1*^{PS2}-tRNA** (0.191 kb)<br>primers: *kpphs1*-tRNA-PS1F/ *kpphs1*-tRNA-PS2R; template: pFC902<br><br>***kpphs1*^{PS2}/*kpade2*^{PS2}-tRNA** (0.191 kb)<br>primers: *kpphs1*-tRNA-PS2F/ *kpade2*-tRNA-PS2R; template: pFC902<br><br>***kpade2*^{PS2}/*kpade2*^{PS5}-tRNA** (0.191 kb)<br>primers: *kpade2*-tRNA-PS2F/ *kpade2*-tRNA-PS5R; template: pFC902<br><br>***kpade2*^{PS5}-tRNA** (0.427 kb)<br>primers: *kpade2*-tRNA-PS5F/ pFC334-R1; template: pFC902	*In vitro*
**pNDH-GDT [pEC0265]** (11.157 kb)	**pNDH-OGG**<br>(Schumacher 2012)<br>digested w/ *Spe*I + *Not*I	**P*gpdA*** (0.969 kb)<br>primers: P*trpC*-hiR/P*gpdA*-ATG-R<br>template: pNAH-GGG [pEC0046] (Erdmann et al. 2022)<br><br>***Dsred*-T*trpC*** (1.364 kb)<br>primers: *Dsred*-P*gpdA*-F/T*trpC*-*niaD*5-R<br>template: pNDH-ADT (Schumacher 2012)	*In vivo*
**pNDN-GDT [pEC0266]** (10.689 kb)	**pNDN-OGG**<br>(Schumacher 2012)<br>digested w/ *Spe*I + *Not*I	**P*gpdA*** (0.969 kb)<br>primers: P*trpC*-hiR/P*gpdA*-ATG-R<br>template: pNAH-GGG [pEC0046] (Erdmann et al. 2022)<br><br>***Dsred*-T*trpC*** (1.364 kb)<br>primers: *Dsred*-P*gpdA*-F/T*trpC*-*niaD*5-R<br>template: pNDH-ADT (Schumacher 2012)	*In vivo*
**pNDG-GDT [pEC0267]** (10.914 kb)	**pNDG-OGG v2**<br>(this study)<br>digested w/ *Spe*I + *Not*I	**P*gpdA*** (0.969 kb)<br>primers: P*trpC*-hiR/P*gpdA*-ATG-R<br>template: pNAH-GGG [pEC0046] (Erdmann et al. 2022)<br><br>***Dsred*-T*trpC*** (1.364 kb)<br>primers: *Dsred*-P*gpdA*-F/T*trpC*-*niaD*5-R<br>template: pNDH-ADT (Schumacher 2012)	*In vivo*
**pNDP-GDT [pEC0268]** (10.671 kb)	**pNDP-OGG**<br>(Erdmann et al. 2022)<br>digested w/ *Spe*I + *Not*I	**P*gpdA*** (0.969 kb)<br>primers: P*trpC*-hiR/P*gpdA*-ATG-R<br>template: pNAH-GGG [pEC0046] (Erdmann et al. 2022)<br><br>***Dsred*-T*trpC*** (1.364 kb)<br>primers: *Dsred*-P*gpdA*-F/T*trpC*-*niaD*5-R<br>template: pNDH-ADT (Schumacher 2012)	*In vivo*
**pNDS-GDT [pEC0269]** (12.606 kb)	**pNDS-OGG v2**<br>(this study)<br>digested w/ SpeI + NotI	**P*gpdA*** (0.969 kb)<br>primers: P*trpC*-hiR/P*gpdA*-ATG-R<br>template: pNAH-GGG [pEC0046] (Erdmann et al. 2022)<br><br>***Dsred*-T*trpC*** (1.364 kb)<br>primers: *Dsred*-P*gpdA*-F/T*trpC*-*niaD*5-R<br>template: pNDH-ADT (Schumacher 2012)	*In vivo*
**pNDH-GNCT [pEC0371]** (11.903 kb)	**pNDH-GDT** (this study)<br>digested w/ *Nco*I + *Not*I	***smh2b*** (0.714 kb)<br>primers: *smh2b*-P*gpdA*-F/*smh2b*-*mch*-R<br>template: pRH2B (Teichert et al. 2014)<br><br>***mch*** (0.794 kb)<br>primers: *mch*-F1/*mch*-NotI-R1<br>template: pNDH-OCT (Schumacher 2012)	*In vitro*

**Name** [ID] (size)	**Entry plasmid**	**Amplicon (s)**		**Method**
**pNDN-GNCT [pEC0372]** (11.435 kb)	**pNDN-GDT** (this study) digested w/ *Nco*I + *Not*I	***smh2b*** (0.714 kb) primers: *smh2b*-P*gpdA*-F/*smh2b*-*mch*-R template: pRH2B (Teichert et al. 2014)	***mch*** (0.794 kb) primers: *mch*-F1/*mch*-NotI-R1 template: pNDH-OCT (Schumacher 2012)	*In vitro*
**pNDG-GNCT [pEC0373]** (11.660 kb)	**pNDG-GDT** (this study) digested w/ *Nco*I + *Not*I	***smh2b*** (0.714 kb) primers: *smh2b*-P*gpdA*-F/*smh2b*-*mch*-R template: pRH2B (Teichert et al. 2014)	***mch*** (0.794 kb) primers: *mch*-F1/*mch*-NotI-R1 template: pNDH-OCT (Schumacher 2012)	*In vitro*
**pNDP-GNCT [pEC0374]** (11.417 kb)	**pNDP-GDT** (this study) digested w/ *Nco*I + *Not*I	***smh2b*** (0.714 kb) primers: *smh2b*-P*gpdA*-F/*smh2b*-*mch*-R template: pRH2B (Teichert et al. 2014)	***mch*** (0.794 kb) primers: *mch*-F1/*mch*-NotI-R1 template: pNDH-OCT (Schumacher 2012)	*In vitro*
**pNDS-GNCT [pEC0375]** (13.352 kb)	**pNDS-GDT** (this study) digested w/ *Nco*I + *Not*I	***smh2b*** (0.714 kb) primers: *smh2b*-P*gpdA*-F/*smh2b*-*mch*-R template: pRH2B (Teichert et al. 2014)	***mch*** (0.794 kb) primers: *mch*-F1/*mch*-NotI-R1 template: pNDH-OCT (Schumacher 2012)	*In vitro*
**pNDH-ONGG [pEC0314]** (11.756 kb)	**pNDH-OGG** (Schumacher 2012) digested with *Nco*I	***smh2b*** (0.712 kb) primers: *smh2b*-P*oliC*-F/*smh2b*-*gfp*-R template: pRH2B (Teichert et al. 2014)	n/a	*In vitro*
**pNDN-ONGG [pEC0315]** (11.288 kb)	**pNDN-OGG** (Schumacher 2012) digested with *Nco*I	***smh2b*** (0.712 kb) primers: *smh2b*-P*oliC*-F/*smh2b*-*gfp*-R template: pRH2B (Teichert et al. 2014)	n/a	*In vitro*
**pNDG-ONGG [pEC0316]** (11.513 kb)	**pNDG-OGG v2** (this study) digested with *Nco*I	***smh2b*** (0.712 kb) primers: *smh2b*-P*oliC*-F/*smh2b*-*gfp*-R template: pRH2B (Teichert et al. 2014)	n/a	*In vitro*
**pNDP-ONGG [pEC0317]** (11.270 kb)	**pNDP-OGG** (Erdmann et al. 2022) digested with *Nco*I	***smh2b*** (0.712 kb) primers: *smh2b*-P*oliC*-F/*smh2b*-*gfp*-R template: pRH2B (Teichert et al. 2014)	n/a	*In vitro*
**pNDS-ONGG [pEC0318]** (13.205 kb)	**pNDS-OGG v2** (this study) digested with *Nco*I	***smh2b*** (0.712 kb) primers: *smh2b*-P*oliC*-F/*smh2b*-*gfp*-R template: pRH2B (Teichert et al. 2014)	n/a	*In vitro*
**pIGR1H-OCG [pEC0403]** (11.566 kb)	**pIGR1H** [pEC0286] (Erdmann et al. 2022) digested w/ *Pac*I	**P*oliC*::*mch*::T*gluc*** (2.116 kb) primers: P*oliC*-*Spe*I-F1/T*gluc*-3RAM-R template: pERG27-OCG (Cohrs et al. 2017)	n/a	*In vivo*
**pIGR1N-OCG [pEC0404]** (11.098 kb)	**pIGR1N** [pEC0288] (Erdmann et al. 2022) digested w/ *Pac*I	**P*oliC*::*mch*::T*gluc*** (2.116 kb) primers: P*oliC*-*Spe*I-F1/T*gluc*-3RAM-R template: pERG27-OCG (Cohrs et al. 2017)	n/a	*In vivo*
**pIGR1G-OCG [pEC0405] (**11.323 kb)	**pIGR1G** [pEC0290] (Erdmann et al. 2022) digested w/ *Pac*I	**P*oliC*::*mch*::T*gluc*** (2.116 kb) primers: P*oliC*-*Spe*I-F1/T*gluc*-3RAM-R template: pERG27-OCG (Cohrs et al. 2017)	n/a	*In vivo*

**Name** [ID] (size)	**Entry plasmid**	**Amplicon (s)**			**Method**
**pIGR1P-OCG**<br>**[pEC0406]** (11.080 kb)	**pIGR1P** [pEC0292]<br>(Erdmann et al. 2022)<br>digested w/ *Pac*I	**P*oliC*::*mch*::T*gluc*** (2.116 kb)<br>primers: P*oliC*-*Spe*I-F1/T*gluc*-3RAM-R<br>template: pERG27-OCG (Cohrs et al. 2017)	n/a		*In vitro*
**pIGR1S-OCG**<br>**[pEC0407]** (13.015 kb)	**pIGR1H** [pEC0286]<br>(Erdmann et al. 2022)<br>digested w/ *Asc*I + *Pac*I	**P*trpC*::*sur*::T*niaD* – v2** (3.270 kb)<br>primers: T*niaD*-5FAM-R/P*trpC*-SpeI-F1<br>template: pNDS-OGG v2 [pEC0307] (this study)	**P*oliC*::*mch*::T*gluc*** (2.116 kb)<br>primers: P*oliC*-*Spe*I-F1/T*gluc*-3RAM-R<br>template: pERG27-OCG		*In vitro*
**pIGR2H-GGT**<br>**[pEC0408]** (12.200 kb)	**pIGR2H** [pEC0287]<br>(Erdmann et al. 2022)<br>digested w/ *Pac*I	**P*gpdA*::*ogfp*** (1.774 kb)<br>primers: P*trpC*-hiR/*ogfp*-T*trpC*-R<br>template: pNAH-GGG [pEC0046] (Erdmann et al. 2022)	**T*trpC*** (0.672 kb)<br>primers: T*trpC*-F1/T*trpC*-3RAM-R<br>template: pNAH-AGT (Schumacher 2012)		*In vivo*
**pIGR2N-GGT**<br>**[pEC0409]** (11.732 kb)	**pIGR2N** [pEC0289]<br>(Erdmann et al. 2022)<br>digested w/ *Pac*I	**P*gpdA*::*ogfp*** (1.774 kb)<br>primers: P*trpC*-hiR/*ogfp*-T*trpC*-R<br>template: pNAH-GGG [pEC0046] (Erdmann et al. 2022)	**T*trpC*** (0.672 kb)<br>primers: T*trpC*-F1/T*trpC*-3RAM-R<br>template: pNAH-AGT (Schumacher 2012)		*In vivo*
**pIGR2G-GGT**<br>**[pEC0410]** (11.957 kb)	**pIGR2G** [pEC0291]<br>(Erdmann et al. 2022)<br>digested w/ *Pac*I	**P*gpdA::ogfp*** (1.774 kb)<br>primers: P*trpC*-hiR/*ogfp*-T*trpC*-R<br>template: pNAH-GGG [pEC0046] (Erdmann et al. 2022)	**T*trpC*** (0.672 kb)<br>primers: T*trpC*-F1/T*trpC*-3RAM-R<br>template: pNAH-AGT (Schumacher 2012)		*In vivo*
**pIGR2P-GGT**<br>**[pEC0411]** (11.714 kb)	**pIGR2P** [pEC0293]<br>(Erdmann et al. 2022)<br>digested w/ *Pac*I	**P*gpdA*::*ogfp*** (1.774 kb)<br>primers: P*trpC*-hiR/*ogfp*-T*trpC*-R<br>template: pNAH-GGG [pEC0046] (Erdmann et al. 2022)	**T*trpC*** (0.672 kb)<br>primers: T*trpC*-F1/T*trpC*-3RAM-R<br>template: pNAH-AGT (Schumacher 2012)		*In vivo*
**pIGR2S-GGT**<br>**[pEC0412]** (13.649 kb)	**pIGR2H** [pEC0287]<br>(Erdmann et al. 2022)<br>digested w/ *Asc*I + *Pac*I	**P*trpC*::*sur*::T*niaD* – v2** (3.270 kb)<br>primers: T*niaD*-5FAM-R/P*trpC*-SpeI-F1<br>template: pNDS-OGG v2 [pEC0307]<br>(this study)	**P*gpdA*::*ogfp*** (1.774 kb)<br>primers: P*trpC*-hiR/*ogfp*-T*trpC*-R<br>template: pNAH-GGG [pEC0046]<br>(Erdmann et al. 2022)	**T*trpC*** (0.672 kb)<br>primers: T*trpC*-F1/T*trpC*-3RAM-R<br>template: pNAH-AGT<br>(Schumacher 2012)	*In vivo*

Table S7 Transformations of *K. petricola* protoplasts carried out in this study.

Strain (alias)	GMO ID	Entry	Donor DNA			CRISPR/Cas9 plasmid
**TET::*gfp*** ^{igr1}	**KP-0460**	WT:A95	**[(T*niaD*::*hph*::P*trpC*)–(P*oliC*::*rtTA*::T*crgA*)–(P*tet*::*gfp*::T*gluc*)]** ^{igr1} (6.522 kb)<br>primers: *kpigr1*-RF-F1/*kpigr1*-RF-R1<br>template: pIGR1H-TGG [pEC0362] (this study)			**pAMA/ribo-*igr1*** ^{PS1}<br>[pEC0058]<br>(Erdmann et al. 2022)
**TET::*gfp*** ^{igr2}	**KP-0256**	WT:A95	**[(T*niiA*::*hph*::P*trpC*)–(P*oliC*::*rtTA*::T*crgA*)–(P*tet*::*gfp*::T*gluc*)]** ^{igr2} (5.845 kb)<br>primers: T*niiA*-*kpigr2*-SH5F/T*gluc*-*kpigr2*-SH3R<br>template: pNAH-OTGG (Janevska et al. 2017)			**pAMA/ribo-*igr2*** ^{PS1}<br>[pEC0059]<br>(Erdmann et al. 2022)
Δpks1/Δphs1/ Δade2	**KP-0282**	WT:A95	**[(T*niaD*::*nat1*::P*trpC*)]** $^{\Delta pks1}$ (1.473 kb); primers: *kppks1*-RT5F/*kppks1*-RT3R; template: pNDN-OGG (Schumacher 2012)	**[(T*niaD*::*hph*::P*trpC*)]** $^{\Delta phs1}$ (1.941 kb); primers: *kpphs1*-RT5F/ *kpphs1*-RT3R; template: pNDH-OGG (Schumacher 2012)	**[(T*niaD*::*bar*::P*trpC*)]** $^{\Delta ade2}$ (1.455 kb); primers: *kpade2*-RT5F/*kpade2*-RT3R; template: pNDP-OGG (Erdmann et al. 2022)	**pAMA/tRNA-*pks1*** ^{PS2}**-*phs1*** ^{PS1}**-*ade2*** ^{PS3}<br>[pEC0195] (this study)
**TET::WCC** ^{black}	**KP-0502**	WT:A95	**[(hygR)–(rtTA)–(P*tet*::*kpwcl1-gfpC*::T*gluc*)]** ^{igr1} (9.311 kb)<br>primers: *kpigr1*-RF-F1/-RF-R1<br>template: pIGR1H-TET::W1GC [pEC0469] (this study)	**[(natR)–(rtTA)–(P*tet*::*gfpN-kpwcl2*::T*trpC*)]** ^{igr2} (7.518 kb)<br>primers: *kpigr2*-RF-F1/-RF-R1<br>template: pIGR2N-TET::GNW2 [pEC0440] (this study)	**[(genR)–(P*gpdA*::*smh2b-mch*::T*trpC*)]** ^{igr3} (4.468 kb) primers: T*niaD*-*kpigr3*-SH5F/T*trpC*-*kpigr3*-SH3R; template: pNDG-GNCT [pEC0373] (this study)	**pAMA/tRNA-*igr1*** ^{PS1}**-*igr2*** ^{PS1}**-*igr3*** ^{PS1}<br>[pEC0467] (this study)
**OE::WCC** ^{black}	**KP-0503**	WT:A95	**[(hygR)–(P*gpdA*::*kpwcl1-gfpC*::T*gluc*)]** ^{igr1} (7.773 kb)<br>primers: *kpigr1*-RF-F1/-RF-R1<br>template: pIGR1H-OE::W1GC [pEC0445] (this study)	**[(natR)–(P*gpdA*::*gfpN-kpwcl2*::T*trpC*)]** ^{igr2} (5.982 kb)<br>primers: *kpigr2*-RF-F1/-RF-R1<br>template: pIGR2N-OE::GNW2 [pEC0446] (this study)	**[(genR)–(P*gpdA*::*smh2b-mch*::T*trpC*)]** ^{igr3} (4.468 kb); primers: T*niaD*-*kpigr3*-SH5F/ T*trpC*-*kpigr3*-SH3R; template: pNDG-GNCT [pEC0373] (this study)	**pAMA/tRNA-*igr1*** ^{PS1}**-*igr2*** ^{PS1}**-*igr3*** ^{PS1}<br>[pEC0467] (this study)
**TET::WCC** ^{rose}	**KP-0484**	WT:A95	**[(hygR)–(rtTA)–(P*tet*::*kpwcl1-gfpC*::T*gluc*)]** ^{pks1} (8.262 kb)<br>primers: T*niaD*-*kppks1*-SH5F/ T*gluc*-*kppks1*-SH3R;<br>template: pIGR1H-TET::W1GC [pEC0469] (this study)	**[(natR)–(rtTA)–(P*tet*::*gfpN-kpwcl2*::T*trpC*)]** ^{phs1} (6.463 kb)<br>primers: T*niaD*-*kpphs1*-SH5F/ T*trpC*-*kpphs1*-SH3R;<br>template: pIGR2N-TET::GNW2 [pEC0440] (this study)	**[(genR)–(P*gpdA*::*smh2b-mch*::T*trpC*)]** ^{ade2} (4.451 kb); primers: *kpade2*-RT5F/ T*trpC*-*kpade2*-SH3R;<br>template: pNDG-GNCT [pEC0373] (this study)	**pAMA/tRNA-*pks1*** ^{PS2}**-*pks1*** ^{PS5}**-*phs1*** ^{PS1}**-*phs1*** ^{PS2}**-*ade2*** ^{PS2}**-*ade2*** ^{PS5}<br>[pEC0389] (this study)
**OE::WCC** ^{rose}	**KP-0485**	WT:A95	**[(hygR)–(P*gpdA*::*kpwcl1-gfpC*::T*gluc*)]** ^{pks1} (6.724 kb)<br>primers: T*niaD*-*kppks1*-SH5F/ T*gluc*-*kppks1*-SH3R;<br>template: pIGR1H-OE::W1GC [pEC0445] (this study)	**[(natR)–(P*gpdA*::*gfpN-kpwcl2*::T*trpC*)]** ^{phs1} (7.525 kb)<br>primers: T*niaD*-*kpphs1*-SH5F/ T*trpC*-*kpphs1*-SH3R;<br>template: pIGR2N-OE::GNW2 [pEC0446] (this study)	**[(genR)–(P*gpdA*::*smh2b-mch*::T*trpC*)]** ^{ade2} (4.451 kb); primers: *kpade2*-RT5F/ T*trpC*-*kpade2*-SH3R;<br>template: pNDG-GNCT [pEC0373] (this study)	**pAMA/tRNA-*pks1*** ^{PS2}**-*pks1*** ^{PS5}**-*phs1*** ^{PS1}**-*phs1*** ^{PS2}**-*ade2*** ^{PS2}**-*ade2*** ^{PS5}<br>[pEC0389] (this study)
**P*oliC*::*gfp*** ^{igr3}	**KP-0426**	WT:A95	**[(T*niaD*::*hph*::P*trpC*)–(P*oliC*::*gfp*::T*gluc*)]** ^{igr3} (4.048 kb)<br>primers: T*niaD*-*kpigr3*-SH5F/T*gluc*-*kpigr3*-SH3R<br>template: pNDH-OGG (Schumacher 2012)			**pAMA/ribo-*igr3*** ^{PS1}<br>[pEC0419]<br>(this study)

Strain (alias)	GMO ID	Entry	Donor DNA		CRISPR/Cas9 plasmid
**P*oliC*::*gfp* *igr4***	**KP-0427**	WT:A95	**[(T*niaD*::*hph*::P*trpC*)–(P*oliC*::*gfp*::T*gluc*)]** *igr4* (4.048 kb)<br>primers: T*niaD-kpigr4*-SH5F/T*gluc-kpigr4*-SH3R<br>template: pNDH-OGG (Schumacher 2012)		**pAMA/ribo-*igr4* PS1**<br>[pEC0420]<br>(this study)
**P*oliC*::*gfp* *igr5***	**KP-0428**	WT:A95	**[(T*niaD*::*hph*::P*trpC*)–(P*oliC*::*gfp*::T*gluc*)]** *igr5* (4.048 kb)<br>primers: T*niaD-kpigr5*-SH5F/T*gluc-kpigr5*-SH3R<br>template: pNDH-OGG (Schumacher 2012)		**pAMA/ribo-*igr5* PS1**<br>[pEC0421]<br>(this study)
**Δ*pks1*/<br>Δ*phs1-phd1***	**KP-0289**	WT:A95	**[(T*niaD*::*bar*::P*trpC*)]** *Δphs1-phd1* (1.455 kb)<br>primers: *kpphs1*-RT5F/*kpphd1*-RT3R<br>template: pNDP-OGG (Erdmann et al. 2022)	**[(T*niaD*::*nat1*::P*trpC*)]** *Δpks1* (1.698 kb)<br>primers: *kppks1*-RT5F/*kppks1*-RT3R<br>template: pNDN-OGG (Schumacher 2012)	**pAMA/tRNA-*pks1* PS2-*phs1* PS1** [pEC0124]<br>(Erdmann et al. 2022)
**TET::*pp***	**KP-0374**	Δ*pks1*/<br>Δ*phs1-phd1*<br>(T1)	**[(T*niaD*::*hph*::P*trpC*)–(P*oliC*::*rtTA*::T*crgA*)–(P*tet*::*kpphs1-2A-kpphd1*::T*gluc*)]** *igr2* (10.835 kb)<br>isolated with *Swa*I from pIGR2H-TET::PP [pEC0355] (this study)		**pAMA/ribo-*igr2* PS1**<br>[pEC0059]<br>(Erdmann et al. 2022)
**TET::*gpp***	**KP-0383**	Δ*pks1*/<br>Δ*phs1-phd1*<br>(T1)	**[(T*niaD*::*hph*::P*trpC*)–(P*oliC*::*rtTA*::T*crgA*)–(P*tet*::*kpgps1-2A-kpphs1-2A-kpphd1*::T*gluc*)]** *igr2*<br>(12.146 kb) isolated with *Swa*I from pIGR2H-TET::GPP<br>[pEC0392] (this study)		**pAMA/ribo-*igr2* PS1**<br>[pEC0059]<br>(Erdmann et al. 2022)
**TET*::pks1***	**KP-0454**	Δ*pks1*/<br>Δ*phs1-phd1*<br>(T1)	**[(T*niaD*::*hph*::P*trpC*)–(P*oliC*::*rtTA*::T*crgA*)–(P*tet*::*kppks1::*T*gluc*)]** *igr2* (13.841 kb)<br>isolated with *Swa*I from pIGR2H-TET::*kppks1*<br>[pEC0394] (this study)		**pAMA/ribo-*igr2* PS1**<br>[pEC0059]<br>(Erdmann et al. 2022)
**Δ*ura3*/<br>TET::*ura3* *igr1***	**KP-0459**	Δ*ura3*<br>(D2)	**[(T*niaD*::*hph*::P*trpC*)–(P*oliC*::*rtTA*::T*crgA*)– (P*tet*::*kpura3*::T*gluc*)]** *igr1* (5.508 kb)<br>primers: T*niaD-kpigr1*-SH5F/T*gluc-kpigr1*-SH3R<br>template: pIGR2H-TET::*kpura3* [pEC0294] (this study)		**pAMA/ribo-*igr1* PS1**<br>[pEC0058]<br>(Erdmann et al. 2022)
**Δ*ura3*/<br>TET::*ura3* *igr2***	**KP-0415**	Δ*ura3*<br>(D2)	**[(T*niaD*::*hph*::P*trpC*)–(P*oliC*::*rtTA*::T*crgA*)–(P*tet*::*kpura3*::T*gluc*)]** *igr2* (8.102 kb)<br>isolated with *Swa*I from pIGR2H-TET::*kpura3*<br>[pEC0294] (this study)		**pAMA/ribo-*igr2* PS1**<br>[pEC0059]<br>(Erdmann et al. 2022)

Supplementary REFERENCES

Cohrs KC, Burbank J, Schumacher J (2017) A new transformant selection system for the gray mold fungus *Botrytis cinerea* based on the expression of fenhexamid-insensitive ERG27 variants. Fungal Genet Biol 100:42-51. https://doi.org/10.1016/j.fgb.2017.02.001

Erdmann EA, Nitsche S, Gorbushina AA, Schumacher J (2022) Genetic engineering of the rock inhabitant *Knufia petricola* provides insight into the biology of extremotolerant black fungi. Front Fungal Biol 3:862429. https://doi.org/10.3389/ffunb.2022.862429

Janevska S, Arndt B, Baumann L, Apken L, Mauriz Marques L, Humpf H-U, Tudzynski B (2017) Establishment of the inducible Tet-On system for the activation of the silent trichosetin gene cluster in *Fusarium fujikuroi*. Toxins 9:126. https://doi.org/10.3390/toxins9040126

Mumberg D, Müller R, Funk M (1994) Regulatable promoters of *Saccharomyces cerevisiae*: comparison of transcriptional activity and their use for heterologous expression. Nucleic Acids Res 22:5767-5768. https://doi.org/10.1093/nar/22.25.5767

Nai C, Wong HY, Pannenbecker A, Broughton WJ, Benoit I, de Vries RP et al. (2013) Nutritional physiology of a rock-inhabiting, model microcolonial fungus from an ancestral lineage of the Chaetothyriales (Ascomycetes). Fungal Genet Biol 56:54-66. https://doi.org/10.1016/j.fgb.2013.04.001

Nødvig CS, Nielsen JB, Kogle ME, Mortensen UH (2015) A CRISPR-Cas9 system for genetic engineering of filamentous fungi. PLoS One 10:e0133085. https://doi.org/10.1371/journal.pone.0133085

Nødvig CS, Hoof JB, Kogle ME, Jarczynska ZD, Lehmbeck J, Klitgaard DK, Mortensen UH (2018) Efficient oligo nucleotide mediated CRISPR-Cas9 gene editing in *Aspergilli*. Fungal Genet Biol 115:78-89. https://doi.org/10.1016/j.fgb.2018.01.004

Schumacher J (2012) Tools for *Botrytis cinerea*: new expression vectors make the gray mold fungus more accessible to cell biology approaches. Fungal Genet Biol 49:483-497. https://doi.org/10.1016/j.fgb.2012.03.005

Teichert I, Steffens EK, Schnass N, Franzel B, Krisp C, Wolters DA, Kück U (2014) PRO40 is a scaffold protein of the cell wall integrity pathway, linking the MAP kinase module to the upstream activator protein kinase C. PLoS Genet 10:e1004582. https://doi.org/10.1371/journal.pgen.1004582

Voigt O, Knabe N, Nitsche S, Erdmann EA, Schumacher J, Gorbushina AA (2020) An advanced genetic toolkit for exploring the biology of the rock-inhabiting black fungus *Knufia petricola*. Sci Rep 10. https://doi.org/10.1038/s41598-020-79120-5

Winston F, Dollard C, Ricupero-Hovasse SL (1995) Construction of a set of convenient *Saccharomyces cerevisiae* strains that are isogenic to S288C. Yeast 11:53-55. https://doi.org/10.1002/yea.320110107
